# Multiple transitions to high l‐DOPA 4,5‐dioxygenase activity reveal molecular pathways to convergent betalain pigmentation in Caryophyllales

**DOI:** 10.1111/nph.70177

**Published:** 2025-05-05

**Authors:** Nathanael Walker‐Hale, M. Alejandra Guerrero‐Rubio, Samuel F. Brockington

**Affiliations:** ^1^ Department of Plant Sciences University of Cambridge Downing Street Cambridge CB2 3EA UK

**Keywords:** anthocyanins, betalains, Caryophyllales, convergent evolution, DODA, pigmentation, specialised metabolism

## Abstract

- Many specialized metabolic pathways have evolved convergently in plants, but distinguishing multiple origins from alternative evolutionary scenarios can be difficult. Here, we explore the evolution of l‐3,4‐dihydroxyphenylalanine (l‐DOPA) 4,5‐dioxygenase (DODA) enzymes to better resolve the convergent evolution of the betalain biosynthetic pathway within the flowering plant order Caryophyllales.
- We use yeast‐based heterologous assays to quantify enzymatic activity of extant proteins and then employ ancestral sequence reconstruction to resurrect and assay ancestral DODA enzymes. We use a combination of ancestral sequence reconstruction, model‐based methods, and structural modelling to describe patterns of molecular convergence.
- We confirm that high l‐DOPA 4,5‐dioxygenase activity is polyphyletic and show that high activity DODAs evolved at least three times from ancestral proteins with low activity. We show that molecular convergence is concentrated proximally to the binding pockets but also appears distally to active sites. Moreover, our analysis also suggests that many unique and divergent substitutions contribute to the evolution of DODA.
- Given the key role of DODA in betalain biosynthesis, our analysis further supports the convergent origins of betalains and illustrates how the iterative evolution of betalain biosynthesis has drawn on a complex mixture of convergent, divergent, and unique variation.

## Introduction

Life is replete with examples of similar traits that have evolved in disparate lineages through convergent evolution (Losos, 2011; Foote *et al*., 2015; Heyduk *et al*., 2019). Widespread phenotypic convergence has been interpreted either as evidence that evolution responds predictably to similar selection pressures, or that evolution is highly constrained in the generation of new variation, or a combination of the two (Losos, 2011). Convergence can therefore be interpreted as evidence of adaptation but also as the result of chance variation which is then fixed by drift (Stayton, 2008, 2015). Investigating the molecular basis of phenotypic convergence offers a powerful framework for exploring the variation and processes driving the iterative evolution of phenotypes (Zhang & Kumar, 1997; Foote *et al*., 2015; Sackton *et al*., 2019). Even distantly related lineages can reuse homologous variation in the evolution of convergent traits, via the reoccurrence of identical or similar mutations, or through different mutations with comparable effects in homologous loci (Christin *et al*., 2010; Stern, 2013; Storz, 2016). By contrast, other studies have revealed convergent phenotypes with a divergent genetic basis, suggesting that either molecular convergence or divergence can be involved in the evolution of convergent traits, or a combination of the two (Hoekstra *et al*., 2006; Natarajan *et al*., 2016; Van Belleghem *et al*., 2023).

Multiple methods have been deployed to distinguish convergence at the molecular level. One approach uses ancestral sequence reconstruction to search for repeated amino acid substitutions at homologous sites, between lineages with convergent phenotypes (Zhang & Kumar, 1997; Castoe *et al*., 2009; Foote *et al*., 2015). Ancestral sequence reconstruction infers all substitutions between parent and descendant states, with repeated amino‐acid substitutions at the same site categorized according to the amino‐acid identity of the parent and descendant states: convergent (if identical descendent states result from parent states that are different), parallel (if identical descendent states result from parent states that are the same), and divergent (if repeated substitutions in the same site result in different descendent states) (Zhang & Kumar, 1997; Castoe *et al*., 2009). Meanwhile, alternative model‐based methods have sought to relax the constraint of amino acid identity to infer adaptive convergence, acknowledging that different states might have similar fitness in the environments of convergent phenotypes (Tamuri *et al*., 2009; Parto & Lartillot, 2018; Rey *et al*., 2019). These model‐based methods also seek to remove false positive inferences derived from neutral convergence, with sites showing evidence of adaptive convergence typically a subset of those inferred by ancestral sequence reconstruction. However, lineage‐specific epistasis might limit the extent to which the same substitution can cause the same functional effect in different backgrounds (Pollock *et al*., 2012; Zou & Zhang, 2015; Storz, 2016). Consequently, ancestral sequence reconstruction coupled to site‐specific mutation of the inferred substitutions in the background of resurrected ancestral proteins (Hochberg & Thornton, 2017) has emphasized that unique and divergent substitutions can be as important as convergent sites for understanding the evolution of protein function (Natarajan *et al*., 2016). Therefore, a combination of these methods is informative in resolving the molecular evolutionary basis of convergent phenotypes.

**Fig. 1:**
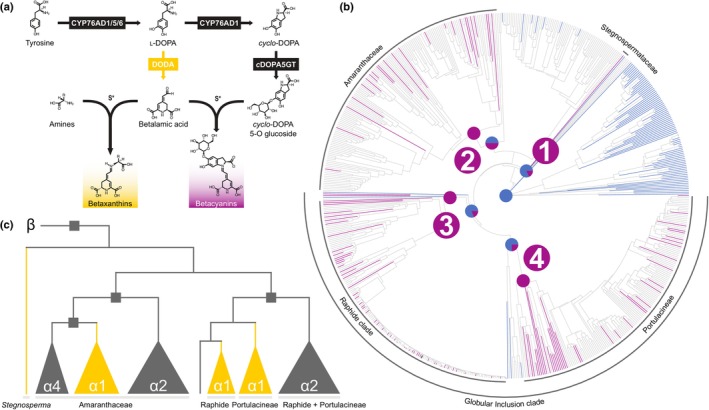
The evolution of betalains in Caryophyllales. (a) The betalain biosynthetic pathway. The transition between l‐DOPA and betalamic acid catalysed by DODA is highlighted in gold. (b) Genus‐level species tree showing the reconstruction of pigmentation states between anthocyanins (blue) and betalains (purple), with four inferred origins highlighted (Sheehan *et al*., 2020). (c) Schematic of the DODA gene tree (Sheehan *et al*., 2020). The root connects to the DODAβ clade, squares at nodes indicate gene duplications, clades are annotated by their designation, and clade colours correspond to l‐DOPA 4,5‐dioxygenase activity of extant sequences: high activity in gold, low/no activity in grey. Clade labels correspond to the arc labels in (b). DODA, l‐DOPA 4,5‐dioxygenase; l‐DOPA, l‐3,4‐dihydroxyphenylalanine.

Plant metabolism features a massive diversity of specialized metabolites which show many striking examples of convergent evolution (Pichersky & Lewinsohn, 2011). Gene duplication and molecular convergence are commonly implicated (Christin *et al*., 2007; Besnard *et al*., 2009; Moghe & Last, 2015), with ancestral sequence reconstruction invaluable in understanding the evolution of enzyme function (Smith *et al*., 2013; Huang *et al*., 2016; Kaltenbach *et al*., 2018; Lichman *et al*., 2020; O'Donnell *et al*., 2021). Betalains are a group of red to yellow pigments which, in flowering plants, occur uniquely in Caryophyllales, where they can replace anthocyanin pigments (Timoneda *et al*., 2019). Previously, it was suggested that betalains could have evolved multiple times (Brockington *et al*., 2011), and we subsequently found further support for up to four transitions with comparative and molecular data (Sheehan *et al*., 2020). A key step in the betalain pathway (Fig. 1a) is the 4,5‐dioxygenase cleavage of l‐3,4‐dihydroxyphenylalanine (l‐DOPA) to form betalamic acid, the core chromophore of betalain pigments (Fig. 1a). In Caryophyllales, this step is accomplished by DODA homologs of the Arabidopsis LigB gene, which exhibit l‐DOPA 4,5‐dioxygenase activity (Christinet *et al*., 2004; Sasaki *et al*., 2009; Chung *et al*., 2015). Brockington *et al*. (2015) showed Caryophyllales‐specific duplications in the DODA gene lineage, resulting in two large clades termed DODAα and DODAβ, with l‐DOPA 4,5‐dioxygenase activity only to be found in the DODAα lineage. Sheehan *et al*. (2020) subsequently showed that l‐DOPA 4,5‐dioxygenase activity was polyphyletic across extant DODAα paralogues. Numerous gene duplications give rise to clades with high l‐DOPA 4,5‐dioxygenase activity, termed DODAα1, and sister clades termed DODAα2 exhibiting little to no l‐DOPA 4,5‐dioxygenase activity (Fig. 1b). The main activity of non‐DODAα1 homologues in Caryophyllales is unknown, although *At*LigB can catalyse 2,3‐cleavage of l‐DOPA to produce muscaflavin and is natively involved in arabidopyrone biosynthesis (Weng *et al*., 2012; Kasei *et al*., 2021). Based on these patterns, Sheehan *et al*. (2020) inferred repeated specialization to betalain pigmentation, underpinned by multiple transitions to high levels of l‐DOPA 4,5‐dioxygenase activity following lineage‐specific gene duplications, consistent with homoplastic patterns of betalain pigment occurrence in extant taxa (Fig. 1c).

Although the evidence provided by Sheehan *et al*. (2020) is compelling, it is inconclusive, and several uncertainties and opportunities remain. First, while we inferred repeated transitions to high l‐DOPA 4,5‐dioxygenase activity, in the absence of ancestral protein resurrection we were unable to rule out a single transition to high activity followed by multiple reversals to low activity. Second, the molecular basis of high l‐DOPA 4,5‐dioxygenase activity in DODAα1‐like sequences, based on a publication by Bean *et al*. (2018) has proved to be controversial (Guerrero‐Rubio *et al*., 2023). Bean *et al*. (2018) compared extant states between *Beta vulgaris* DODAα1 and DODAα2 proteins at sites that were consistently different in the context of a broader sample of Caryophyllales DODAs. By mutating these sites in DODAα2 to match amino acid states found in DODAα1, they suggested that only seven mutations were responsible for the evolution of high activity. However, we were subsequently unable to reproduce their result (Guerrero‐Rubio *et al*., 2023). Although some of the sites implicated by Bean *et al*. (2018) seem good candidates based on comparative analysis (Sheehan *et al*., 2020; Guerrero‐Rubio *et al*., 2023), the fact that these residues are not sufficient to confer high activity on DODAα2 suggests further undescribed evolutionary complexity. The concept of convergent betalain pigmentation is increasingly evidenced (Sheehan *et al*., 2020; Pucker *et al*., 2024) but has not yet been leveraged to yield insight into this complexity. Consequently, the evolutionary path(s) to high activity remain unknown, and the degree of molecular convergence between different transitions to high activity is unexplored.

Here, we interrogate the molecular evolution of high l‐DOPA 4,5‐dioxygenase activity in Caryophyllales, considering the multiple origin hypothesis. First, we curate a dense array of extant DODAα homologs and assay their activity in yeast, confirming the polyphyletic distribution of high activity. We infer a DODAα gene tree, reconciled to the Caryophyllales species phylogeny, and use it to reconstruct ancestral sequences. We resurrect these ancestral sequences in yeast and show that high activity evolved three times. We then infer historical substitutions, and by investigating their structural context, find a mixture of convergent, divergent, and unique substitutions implicated in the evolution of high activity. We demonstrate that adaptive convergent evolution of DODA enzyme structure and function occurs within a complex sequence landscape, shedding new light on the intricate molecular evolution of betalains in Caryophyllales.

## Materials and Methods

### Phylogenetic trees

We started from the most inclusive sample of DODAα sequences from Sheehan *et al*. (2020), derived from a mixture of genome and transcriptome assemblies. We aimed to infer a reconciled gene tree with nodes identified as speciation or duplication events. To do so, we required a species tree matching our DODA sampling. We started with the maximum quartet support species tree from Walker *et al*. (2018) and expanded it to sampled taxa not present in that tree. Because the non‐overlapping taxa almost always had one or more congenerics in the tree, we did not consider it necessary to conduct a full phylogenomic analysis from scratch. Instead, we added taxa by surveying the literature for subtrees that included their relationships relative to taxa already included. If no such tree was available, we used PyPHLAWD (Smith & Walker, 2019) to scrape sequence data for the genus of the missing taxon from the NCBI (‘pln’ database, downloaded 29/11/2020), used PyPHLAWD's find_good_clusters.py to create a concatenated alignment and inferred a maximum likelihood tree from the nucleotide data with IQ‐Tree v.2.1.2 (Minh *et al*., 2020) under the Generalised Time Reversible model with empirical frequencies and discrete gamma‐distributed among‐site rate variation with four categories (GTR + F + G4) model, before adding the taxon to the overall tree based on the relationships in the genus tree. Finally, if no data was available from the NCBI, we proceeded from the assembled transcriptomes used in Sheehan *et al*. (2020) for the genus of the taxon and a single outgroup chosen based on the relationships in the overall tree, and inferred one‐to‐one orthologues with the pipeline from Yang & Smith (2014). Amino acid sequences for the resulting orthologues were aligned with mafft v.7.453 (‐‐auto; Katoh & Standley, 2013) and sparse columns were removed with pxclsq from phyx v.1.3.1 (‐p 0.1; Brown *et al*., 2017). The resulting alignments were concatenated with pxconcat from phyx and a maximum likelihood tree was inferred with IQ‐Tree with the edge‐linked proportional model (‐spp) under GTR + F + G4. Finally, the missing taxon was placed into the overall tree based on the relationships in the smaller tree. The resulting species tree is shown in Supporting Information Fig. S1.

The collection of DODAα coding sequences was aligned with prank v.170427 under the empirical codon model (‐codon ‐iterate = 5; Löytynoja & Goldman, 2005), sparse columns were removed with pxclsq (‐p 0.1), and the resulting alignment was used for maximum likelihood gene tree reconciliation with GeneRax v.1.2.2 (Morel *et al*., 2020) under GTR + F + G4 and the UndatedDL model. The resulting maximum likelihood reconciliation featured a deep duplication that was poorly supported by taxon sampling and implied large and unparsimonious losses (Fig. S2), so we therefore further constrained this tree to match the species tree by placing *Limeum aethiopicum* DODAα sister to the Globular Inclusion clade and *Stegnosperma halimifolium* DODAα1 sister to *S. halimifolium* DODAα2, creating a species‐specific duplication. The resulting tree is shown in Fig. S3.

### Ancestral sequence reconstruction

The full DODAα alignment contained multiple fragmentary sequences derived from transcriptome assemblies, which we reasoned would create challenges for ancestral sequence reconstruction with gaps. We therefore winnowed this alignment to remove partial sequences by removing sequences with long terminal deletions and removing sequences with internal insertions or deletions longer than two amino acids if they were not conserved in at least two sequences. We retained a truncated transcript of DODAα from *Microtea debilis* as it was the only sequence available from Microteaceae. We pruned the reconciled gene tree to match this reduced sampling (Fig. S4), and the reduced alignment was used to optimise branch lengths under GTR + F + G4 with raxml‐ng v.1.0.1 (‐‐evaluate ‐‐lh‐epsilon 0.001; Kozlov *et al*., 2019). The resulting tree was used as a guide tree for a second round of prank alignment with the reduced sequence set under the empirical codon model (‐codon ‐t = [reduced tree]). The resulting in‐frame alignment was translated to amino acids and used to optimise amino acid branch lengths on the reduced topology with RAxML‐NG under the best‐fitting model of amino acid sequence evolution, Jones‐Taylor‐Thornton with discrete gamma‐distributed among‐site rate variation with four categories (JTT + G4; ‐‐evaluate ‐‐model JTT + G4 ‐‐lh‐epsilon 0.001). Finally, FastML v.3.11 (Pupko *et al*., 2000, 2002) was used to reconstruct ancestral amino acid sequences with marginal inference under JTT + G4. Adjacent gaps were amalgamated into individual characters and reconstructed as binary presence‐absence with stochastic mapping, preferring gap over sequence if the Posterior Probability (PP) > 0.5. For each node, we extracted the *Maximum A Posteriori* (MAP) sequence. To account for reconstruction uncertainty, we also calculated the AltAll sequence (Eick *et al*., 2016) which replaces the MAP state at ambiguous sites with the second most probable state if PP > 0.2. We left reconstructed gap states fixed between MAP and AltAll. Two nodes in our tree were separated by a zero‐length amino acid branch length, and the inferred marginal distributions were identical; we therefore carried forward a single sequence to represent these nodes.

### Yeast expression

Sequences inferred in the ancestral reconstruction of DODA enzymes were synthetically obtained from Twist Bioscience (San Francisco, CA, USA) and expressed in *Saccharomyces cerevisiae* to test their betalain‐production ability. Gene expression was conducted by using Golden Gate Assembly and the yeast toolkit described by Lee *et al*. (2015). This procedure involved two main steps where the coding sequences were firstly expressed in the entry vector pYTK001 and then transferred to integration vectors. All DODA sequences were codon optimized for their expression in *S. cerevisiae* and domesticated to remove restriction sites employed in this protocol: the recognition sites for the restriction enzymes *Bsm*BI and *Bsa*I were eliminated for the cloning step and the recognition sites for NotI were removed to facilitate genomic integration. The strain yHS023, previously created in our laboratory (Guerrero‐Rubio *et al*., 2023), was employed as a backbone for the expression of DODA sequences in the LEU locus. The strain yHS023 is a modified version of BY4741 (MATa his3Δ1 leu2Δ0 met15Δ0 ura3Δ0) that expresses the coding sequence of CYP76AD6 from Beta vulgaris (GenBank ID: OQ362268.1) in the URA3 locus. BvCYP76AD6 is a CYP76AD1 homologue capable of hydroxylating tyrosine to l‐DOPA (Sunnadeniya *et al*., 2016), and yeast strains expressing this in combination with a DODA homologue capable of l‐DOPA 4,5‐dioxygenase activity will produce yellow betaxanthins. Thus, the DODA sequences were cloned into the LEU2 integration vector pTMP137 (provided by J. E. Dueber, University of California, Berkeley), with the promoter ScCCW12 and the terminator ScADH1. Genomic integration of linearized plasmids was performed by digestion with NotI and yeast transformation using the high‐efficiency LiAc/SS carrier DNA/PEG yeast transformation protocol (Gietz & Schiestl, 2007). Cells were grown on complete supplement media lacking uracil and leucine for positive colonies selection. All plasmids were verified by Sanger sequencing and restriction digest. Primers used in this work are shown in Table S1. All the strains constructed in this work are listed in Table S2.

To quantify the production of betaxanthins as a proxy for l‐DOPA 4,5‐dioxygenase activity, yeast strains were grown in 2 ml of complete supplement media lacking uracil and supplemented with 1 mM tyrosine and 10 mM ascorbic acid. The strain yHS023 was employed as a negative control. All samples were grown at 30°C, 180 rpm orbital shaking, and after 48 h, 10 μl of saturated cultures were diluted in 490 μl of fresh medium and placed into deep 96‐well plates. The samples were incubated for 24 h at 30°C, 1000 rpm orbital shaking. Then, cells were centrifuged for 2 min at 4000 rpm (2343 ***g***) and resuspended in phosphate‐buffered saline (PBS) pH 7.4. After two rounds of washing, 100 μl of PBS containing cells was used to quantify intracellular betaxanthin levels using a ClarioStar microplate reader (BMG LABTECH, UK). Fluorescence of betaxanthins was detected by using excitation wavelength 470 nm and emission wavelength 510 nm. Values were normalized based on the negative control strain (yHS023) and corrected by the cell density (OD600). We conducted four replicates per strain.

### Inferring historical substitutions

Historical substitutions were inferred by compiling sequence differences between ancestral and descendant MAP sequences along a branch, or the ancestor and final descendant of a multibranch lineage. For a set of branches or lineages of interest, we generated all two‐way combinations and then filtered any that were sister or were a direct ancestor or descendant of one another. Overlapping substitutions for each pair were compiled and defined as divergent if they resulted in a different state or convergent if they resulted in the same state. Unique substitutions for each branch that did not overlap between any pair were also compiled. Utilities for this purpose were implemented in the scripts count_conv_subs_from_asr.py and get_conv_subs_from_asr.py, available from https://github.com/NatJWalker‐Hale/convergence_tools.

### Expected number of molecular convergences

To generate an expectation for neutral convergence, we adopted the approach of Zou & Zhang (2015). Posterior site rates under JTT + G4 were inferred on the reconciled tree with paml v.4.9h (Yang, 1997). Site‐specific frequencies were calculated from the alignment and smoothed under a Jeffreys prior by adding a pseudocount of 0.5 to each character. For each branch combination consisting of parents $X_{1}$ and $X_{2}$ and descendants $X_{3}$ and $X_{4}$ and for each site, the MAP character at the Most Recent Common Ancestor (MRCA) node of the two focal branches, $X_{0}$, was extracted from the FastML result. If the MAP character is amino acid j, $P\left(X_{0}\right)=I_{j}$, a vector that is 1 for amino acid j and 0 otherwise. For the parent node of each focal branch, the probability of observing each amino acid conditional on $P\left(X_{0}\right)$ is given by $P\left(X_{1}|X_{0}=j\right)=I_{j}\cdot e^{Qt_{1}r}$, and $P\left(X_{2}|X_{0}=j\right)=I_{j}\cdot e^{Qt_{2}r}$, where Q is the instantaneous rate matrix calculated from JTT exchangeabilities from paml, combined with the smoothed site‐specific frequencies and scaled so that the average rate of transition is one in the usual manner (Yang, 2014), $t_{k}$ is the sum of the branch lengths separating the MRCA node and the parent node $X_{k}$ of the focal branch, in substitutions per site, r is the posterior rate for that site under JTT + G4 from paml, and e signifies the matrix exponential. Conditional on the amino acid appearing at the parent node, $P\left(X_{3}|X_{1}=j\right)=I_{j}\cdot e^{Qt_{3}r}$ and $P\left(X_{4}|X_{2}=j\right)=I_{j}\cdot e^{Qt_{4}r}$. Then, the joint probability of amino acids A, B, C, and D at nodes $X_{1}-X_{4}$ is $P\left(A,B,C,D\right)=P\left(X_{1}=A\right)\cdot P\left(X_{3}=C|X_{1}=A\right)\;\cdot P\left(X_{2}=B\right)\cdot P\left(X_{4}=D|X_{2}=B\right)$. Finally, we calculated the probability of a convergent substitution at the two parent nodes and two descendant nodes with states A, B, C, and D as $P_{\text{conv}}=\sum_{A\neq C,B\neq D,C=D}P\left(A,B,C,D\right)$ (Zou & Zhang, 2015). The probability of a divergent substitution was calculated similarly. These quantities were summed over all sites not inferred or observed to contain gaps in any of the relevant nodes to generate an expectation for the alignment. If the MRCA of the parents of two focal branches was one of the parents, we calculated $P\left(X_{0}\right)$ one node deeper in the tree. Expected values were compared to observed counts as the ratio observed/expected, and a *P*‐value was calculated from a Poisson distribution with the expected value as the rate parameter, right tailed for ratio > 1 and left tailed for ratios < 1.

We conducted a similar analysis with parametric bootstrapping by simulation. Individual sites were simulated along the reconciled tree with seq‐gen v.1.3.4 under JTT, using the smoothed site‐specific frequencies and scaling branch lengths in the tree with the posterior rate per site from paml and recording ancestral sequences. Sites were concatenated and gaps were cloned from the reconstructed sequences into each simulated alignment. We simulated as many sites as the original alignment (789), and repeated this procedure for 1000 replicate simulated alignments. Convergences and divergences were counted for each branch pair as above, and an expectation was generated by dividing the total count over all replicates by the number of replicate alignments. A *P*‐value was calculated by computing the proportion of replicates with a result greater than or equal to (or less than or equal to) the observed value. Both approaches are implemented in python in the scripts calc_expected_conv.py and sim_expected_conv.py, available from https://github.com/NatJWalker‐Hale/convergence_tools.

### Detecting adaptive amino acid convergence

To infer adaptive convergence in sites, we applied a suite of methods previously used to infer adaptive convergence (Rey *et al*., 2019) or detect shifts in directional selection (Duchemin *et al*., 2023): the branch‐site test with classic codon models (Zhang *et al*., 2005; Christin *et al*., 2007) as implemented in Godon v.0.8 (Davydov *et al*., 2019); pcoc v.1.0.1, a method to detect convergent substitutions and site‐specific amino acid frequency shifts (Rey *et al*., 2018); pelican v.1.0.8, a method to detect site‐specific amino acid frequency shifts (Tamuri *et al*., 2009; Duchemin *et al*., 2023); and diffsel (https://github.com/vlanore/diffsel), a Bayesian method in the MutSel framework to detect differential amino acid fitness effects (Parto & Lartillot, 2017, 2018). For all methods, the same alignment and reconciled gene tree used for ancestral sequence reconstruction was used. The alignment was first pruned of sparse sites using pxclsq (‐p 0.1). Based on the activities of reconstructed proteins, foreground branches were specified as: the tip leading to *S. halimifolium* DODAα1; the branch subtending the Amaranthaceae *s. l*. DODAα1 and DODAα4 clade and all descendant branches but excluding the branch subtending DODAα4 and all descendant branches; and the branch subtending the Globular Inclusion clade DODAα1 and all descendant branches, excluding *K. caespitosa* DODAα1. For the branch site test, only the first and immediate descendant branches were used as foreground, and all foreground branches were tested simultaneously. The branch‐site test was run without non‐synonymous rate variation and a significantly improved fit over the null was determined with a Likelihood Ratio Test on 1 degree of freedom. PCOC was run with C60 profiles and gamma‐distributed among‐site rate variation, allowing at most 10% gaps in all and in foreground sites. Diffsel was run with two chains for 3000 generations and a 20% burn‐in, using the tracecomp utility to determine that all effective sample sizes were > 200 and chain differences < 0.1. We calculated a score between 0 and 1 for each method (Rey *et al*., 2019): the Bayes Empirical Bayes (BEB) Posterior Probability of a positively selected site for the branch‐site test, the score for the PCOC model, 1 minus the *P*‐value for the site for PELICAN, and for diffsel a transformation of the posterior probability that the log‐ratio of amino acid fitness is > or < 0, such that high probabilities of substantial positive or negative fitness differences between foreground and background branches score close to 1.

### Protein structure, divergence, and conservation

Protein structures were predicted using AlphaFold2 as implemented in colabfold (AlphaFold2_mmseqs; https://github.com/sokrypton/ColabFold, Jumper *et al*., 2021; Mirdita *et al*., 2022). P2rank v.2.4 was used to predict binding pockets with the AlphaFold configuration (‐c alphafold; Krivák & Hoksza, 2018). Structures were visualised in ChimeraX v.1.7.1 (Meng *et al*., 2023). We aimed to generate a metric that summarised per‐site divergence between DODAα1‐like and DODAα2‐like sequences as well as their relative conservation within each class. To do this, we calculated the per‐site Jensen‐Shannon Divergence (JSD) between DODAα1 and DODAα2‐like sequences in each clade using the script calc_site_specific_divergence_aa_n_aln.py, and calculated per‐site conservation as 1 minus the normalised Shannon entropy, such that fixed sites have a value of 1, using the script calc_site_specific_aa_var.py. Both scripts are available from https://github.com/NatJWalker‐Hale/alignment_and_tree_tools. Both values range from 0 to 1; to create a combined metric we calculated the mean normalised entropy for each DODAα1‐DODAα2 comparison and multiplied it by the JSD. Thus, values of 1 correspond to sites that are fixed on different amino acids in each group. In each case, we used the clade(s) of DODAα1 and DODAα2‐like sequences belonging to a specific taxonomic group (i.e. for Amaranthaceae, we compared DODAα1 sequences to DODAα2 and DODAα4 sequences; for Portulacineae, we compared Portulacineae DODAα1 sequences to Portulacineae DODAα2 sequences, etc.).

## Results

### Polyphyletic distribution of high l‐DOPA 4,5‐dioxygenase activity across the DODA tree

**Fig. 2:**
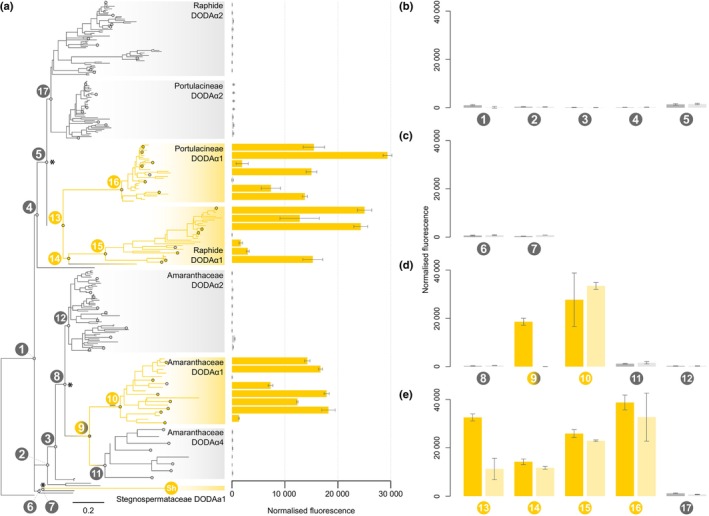
Multiple transitions to high l‐DOPA 4,5‐dioxygenase activity. (a) Reconciled gene tree of DODAα with the ancestral nodes characterized in this work are labelled numerically. Grey branches are inferred with low/no l‐DOPA 4,5‐dioxygenase activity, and gold branches with high activity. Asterisks at nodes indicate major gene duplication events. Circles at tips show characterised extant sequences with their betaxanthin fluorescence measured from heterologous expression in yeast is shown as horizontal bar charts. Asterisks at terminals within Portulacineae DODAα1 denote measured fluorescence lower than background. (b–e) Measured betaxanthin fluorescence following expression of ancestral resurrected sequences in yeast. Darker bars (left hand side for each node) correspond to the MAP sequence, and lighter bars to the AltAll. Node numbers correspond to those depicted in (a). (b) Depicts the backbone nodes. (c) Stegnospermtaceae origin. (d) Amaranthanceae origin. (e) Globular Inclusion (including the Raphide and Portulacineae clades) origin. Bar heights are means, error bars ±1 SD. DODA, l‐DOPA 4,5‐dioxygenase activity; l‐DOPA, l‐3,4‐dihydroxyphenylalanine.

We used a Caryophyllales species tree and a diverse sample of DODA sequences from across Caryophyllales to infer a reconciled gene tree of the DODAα clade (Fig. 2a). We took a sample of full‐length sequences from all major DODAα clades in the tree and expressed them in yeast in combination with a CYP76AD6 homolog capable of hydroxylating tyrosine to form l‐DOPA. The ability of transformed colonies to produce yellow betaxanthins serves as a proxy for l‐DOPA 4,5‐dioxygenase activity. The results confirmed a polyphyletic distribution of activity in DODAα, with multiple high activity clades sister to clades with low to no activity (Fig. 2a; Sheehan *et al*., 2020). Measured activities did not show obvious clade‐specific patterns or optima and was highly variable among all characterized sequences (Fig. 2a). Three DODAα1‐like sequences showed extremely low activity, one of which, in *Spinacia oleracea*, appears to be explained by loss of activity following a shallow, species‐specific tandem gene duplication (Figs 2a, S5). The other two examples (*Rivina humilis* DODAα1 and *Pterocactus tuberosus* DODAα1) could also be plausibly explained by a similar pattern, but because our sequence data for these species are derived from transcriptomes, we were unable to determine if their genomes encode additional paralogs with high activity.

### High l‐DOPA 4,5‐dioxygenase activity evolved three times in Caryophyllales

We then reconstructed sequences at internal nodes using marginal inference, summarizing the posterior probability distribution of states at each site using both a *Maximum A Posteriori* (MAP) sequence and an AltAll sequence in which we replaced ambiguous states with the second highest probability state if it had PP > 0.2 (Eick *et al*., 2016). All nodes had average maximum site‐state PP > 0.95, suggesting confident inference (Fig. S6). MAP and AltAll sequences were synthesized and expressed in yeast with a CYP76AD6 homolog to provide the l‐DOPA substrate and assessed for betaxanthin production. The results support the inference that high l‐DOPA 4,5‐dioxygenase activity evolved multiple times, with high activity sequences evolving three times independently from low activity ancestors following gene duplication (Fig. 2b–e). Multiple nodes along the backbone of DODAα show little or no activity (nodes 1–5; Fig. 2b). The ancestors to *Macarthuria australis* and *S. halimifolium* DODAα1 show low activity (nodes 6, 7; Fig. 2c), and while we were unable to express *S. halimifolium* DODAα1 in yeast, our previous *in planta* expression data shows it to possess high activity (Sh, Fig. 2a; Sheehan *et al*., 2020) indicating a separate origin in *Stegnosperma*. Taxonomically, therefore, three origins occur: within the Stegnospermataceae, the Amaranthaceae, and the Globular Inclusion clade (Fig. 2).

### Patterns of evolving l‐DOPA 4,5‐dioxygenase activity within origins

Having established multiple separate transitions to high l‐DOPA 4,5‐dioxygenase activity, we then sought to characterize patterns of activity within each origin by characterizing nodes descended from each inferred initial transition. We observed that the inferred transition to high activity in Amaranthaceae differed depending on the MAP or AltAll sequence. The MAP sequence implies a transition in the ancestral sequence of DODAα1 and DODAα4 (Fig. 2d, node 9), with a subsequent loss of high activity in DODAα4. However, the AltAll sequence implies that this node lacked high activity, instead suggesting this transition occurred in the ancestor of DODAα1 (Fig. 2d, node 10). In the Globular Inclusion clade, the ancestor of DODAα1 shows roughly a third of the activity in the AltAll sequence than the MAP (Fig. 2e, node 13), although both are far greater than the ancestral low activity nodes. In both Amaranthaceae and Globular Inclusion lineages, descendant nodes seem to show elevated levels of activity relative to the initial transition node (Fig. 2d,e, node 9 vs node 10, node 13 vs nodes 14–16). However, due to difficulties in interpreting the cause of quantitative differences in heterologous expression data, we avoided attempting to further characterize this (see the Discussion section).

### Convergent, divergent, and unique substitutions during transitions to high DODA

**Fig. 3:**
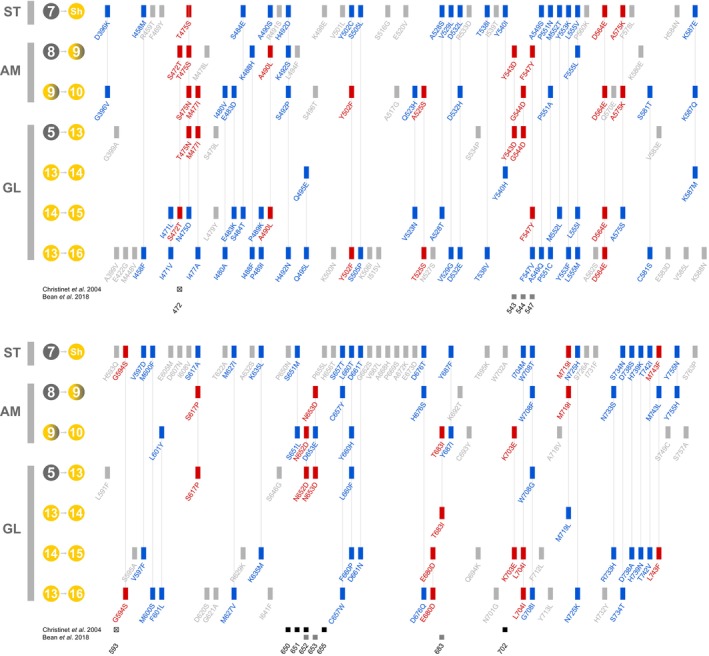
Convergent, divergent, and unique substitutions during transitions to high l‐DOPA 4,5‐dioxygenase activity. Sites are numbered according to their position in our alignment. The figure is divided into two halves with the top half representing the 5′ end of the alignment (positions 396–588) and the bottom half representing the 3′ end of the alignment (positions 591–763). Branches and nodes are assigned to each of the three origins: Stegnospermataceae (ST), Amaranthaceae (AM) and Globular Inclusion (GL). Areas outside of these parts of the alignment show no substitutions on the branches in question and so are not depicted. Branches implicated in the transitions to high l‐DOPA 4,5‐dioxygenase activity are labelled according to their parent–daughter nodes. Overlapping substitutions between at least two evolutionary branches are classified as convergent (red) if they result in the same state and divergent (blue) if they occur in the same position but result in different states. Unique non‐overlapping substitutions on each branch are shown in grey. At the bottom, overlaps between sites containing inferred substitutions in our alignment and sites previously implicated by Christinet *et al*. (2004) (crossed box, substrate or cofactor orienting; filled box, betalain taxon motif) and Bean *et al*. (2018) in the evolution of DODA function are highlighted. DODA, l ‐DOPA 4,5‐dioxygenase; DOPA, l‐3,4‐dihydroxyphenylalanine.

We next investigated whether independent transitions to high activity involved convergent molecular evolution. By comparing ancestral and descendant sequences across multiple branches associated with each inferred transition to high activity, we tracked historical substitutions for each branch and identified shared patterns. For each possible pair of independent branches, we cataloged overlapping substitutions, categorizing them as follows: *convergent substitutions*, where the descendant amino acids at a given residue position were identical; *divergent substitutions*, where substitutions occurred at the same residue but resulted in different amino acids; and *unique substitutions*, found in only one branch within any given comparison. The results of this analysis are summarized in Fig. 3. In the *Stegnosperma* origin, we identified 75 substitutions, of which 6 (8%) were convergent with other origins, 37 (50%) were divergent, and 32 (42%) were unique. For the Amaranthaceae origin, 52 substitutions were identified: 18 (35%) were convergent, 23 (44%) were divergent, and 11 (21%) were unique. In the Globular Inclusion origin, we inferred 100 substitutions, with 22 (22%) convergent, 50 (50%) divergent, and 28 (28%) unique. Across all branch comparisons between different origins, we identified 64 sites with either convergent or divergent substitutions. Only 2 sites (3%) exhibited convergence across all three origins (T475S and D564E); 21 sites (33%) had a convergent substitution in at least two origins, while 42 sites (66%) were exclusively divergent across at least two origins. In 11 site‐branch combinations (17%), substitutions were convergent between two origins but divergent in the third. Furthermore, we observed that, in cases involving sequential branches associated with evolving high DODA, certain sites (e.g. 475 and 653) displayed complex patterns, with a convergent substitution in one branch followed by a divergent substitution in the subsequent branch, or vice versa (Fig. 3). Some of the sites containing convergent substitutions matched those previously implicated in controlling DODA biochemical function (Fig. 3; Christinet *et al*., 2004; Bean *et al*., 2018; Chiang *et al*., 2025).

### Statistical tests detect excess convergence among origins and key transition branches

**Fig. 4:**
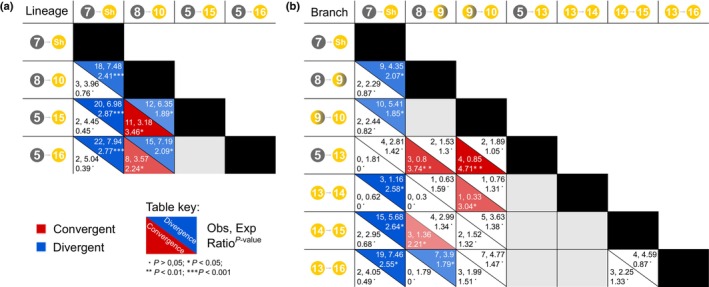
Excess convergence and divergence among different origins. Branch pairs that are not sister or directly ancestral or descended are compared across different major lineages of DODAα1 (Stegnospermataceae, Amaranthaceae, Raphide Clade and Portulacineae). Each cell gives the observed value, expected value, ratio, and *P*‐value from the Poisson distribution with rate equal to the expected value. The lower half p gives convergence, upper half gives divergence. Significant convergent results are highlighted in red and significant divergent results in blue. (a) Results from multi‐branch lineages and (b) results from branches. DODA, l‐3,4‐dihydroxyphenylalanine (l‐DOPA) 4,5‐dioxygenase.

Notably, the proportion of inferred molecular convergence per origin, expressed as a percentage of substitutions, is relatively low, ranging from 8% to 35%. Given some neutral convergence is expected due to the limited number of amino acid states and random drift, we adapted a method from Zou & Zhang (2015) to determine whether the observed levels of convergence or divergence exceeded those predicted by the best‐fitting empirical model of amino acid sequence evolution. By accounting for site‐rate heterogeneity and amino acid frequency variation, we found that convergence between the Amaranthaceae DODAα1 lineage (node 8 to node 10) and both Globular Inclusion clade DODAα1 lineages (node 5 to node 15 and node 5 to node 16; Fig. 4a) was significantly higher than expected. However, we did not observe excess convergence between these lineages and Stegnospermataceae DODAα1, instead finding a greater‐than‐expected number of divergent substitutions within the Stegnospermataceae origin (node 7 to Sh, Fig. 4a). Upon further dissecting lineages into constituent branches, we identified a more intricate pattern of convergence and divergence (Fig. 4b). For example, both branches within the Amaranthaceae DODAα1 lineage (node 8 to node 10) exhibited greater‐than‐expected convergence with the initial branch within the Globular Inclusion DODAα1 transition, although only the initial Amaranthaceae branch converged with the Raphide branch of DODAα1 (node 8 to node 9 vs node 5 to node 13/node 14 to node 15, Fig. 4b). Additionally, the branch subtending Amaranthaceae DODAα1 showed an enriched convergence with the branch subtending Raphide and K. caespitosa DODAα1 (node 13 to node 14, Fig. 4b), though this enrichment may be artifactual, driven by a single convergent substitution and a short branch length. We also applied an analogous approach with a parametric bootstrapping approach through simulation. This analysis identified fewer lineage and branch pairs with significantly higher‐than‐expected convergence. Nonetheless, the Amaranthaceae DODAα1 lineage retained significant convergence with the Raphide DODAα1 lineage, and the initial branch in Amaranthaceae DODAα1 was significantly enriched for convergence with the initial branch within the Globular Inclusion clade DODAα1 (Fig. S7). Notably, the *S. halimifolium* DODAα1 branch demonstrated higher‐than‐expected divergence relative to all other origins, albeit with only select individual branches within these origins (Fig. S7).

### Model‐based methods identify a variable suite of adaptive convergent residues

**Fig. 5:**
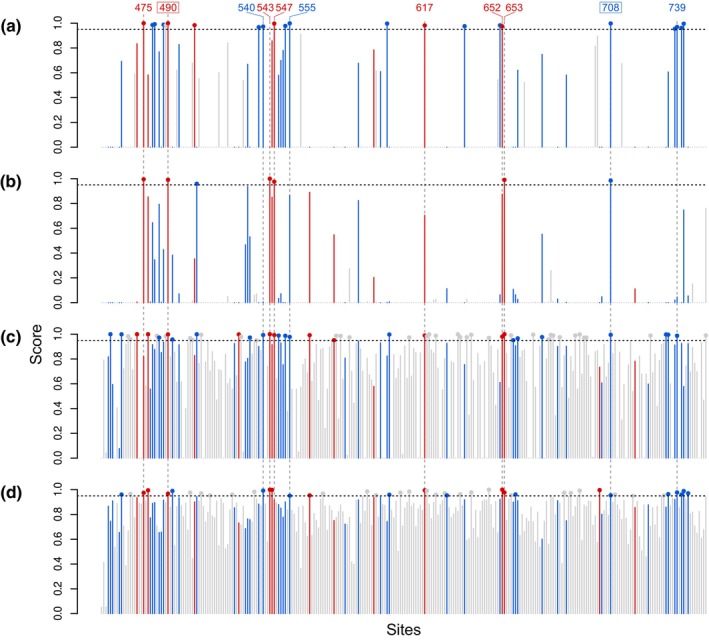
Site‐based methods show evidence for adaptive convergence. Results from a suite of methods to detect adaptive convergence on the 274 amino acid sites in the cleaned alignment. The four methods are: (a) branch‐site Bayes Empirical Bayes (BEB), (b) PCOC, (c) PELICAN, and (d) diffsel. Sites corresponding to convergent substitution identified by ancestral sequence reconstruction are coloured red, and sites corresponding to divergent substitution are coloured blue. Sites implicated by all four methods are shown in bordered text, while sites implicated by three out of four are shown by text only. Numbered positions correspond to the full alignment. Score is calculated differently for each method, see the Materials and Methods section for details.

Building on the evidence for adaptive convergence across entire branches or origins, we applied a suite of methods to identify adaptive convergence at individual residue sites. Using four distinct approaches – branch‐site BEB, PCOC, PELICAN, and Diffsel – we detected strong evidence of adaptive convergence at multiple sites. Many of these sites also showed convergent and divergent substitutions in the branches of interest, as identified by our ancestral sequence reconstruction analyses (Fig. 5a–d). Each method exhibited varying sensitivity: with a score threshold of 0.95, branch‐site BEB identified 22 convergent residues, PCOC detected 7, PELICAN flagged 62, and Diffsel identified 47. Only two sites were consistently supported across all four methods, with an additional nine sites supported by three out of four methods. All of these sites showed at least one convergent or divergent substitution, with several aligning with previously identified residues critical to DODA function (Figs 3, 5).

### Structural insights into the functional significance of inferred substitutions

**Fig. 6:**
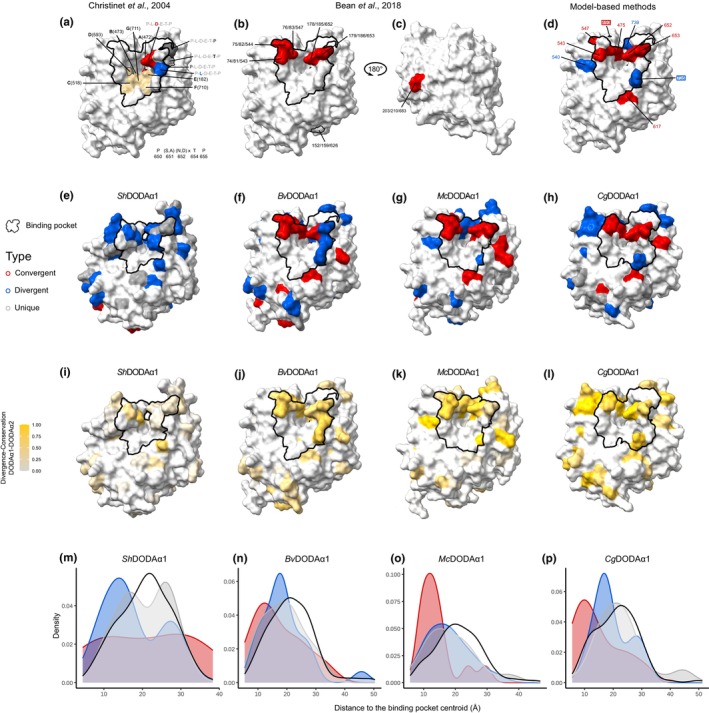
Convergent and divergent substitutions are concentrated around the DODA binding pocket. Predicted structures of four extant representative DODAα1 proteins, with sites implicated in enzyme function highlighted. In each case homologous residues are identified in our alignment. Except where otherwise specified, residues are coloured according to if they are inferred to contain a substitution along any focal branch(es): red, convergent; blue, divergent; dark grey, unique. Each structure shows the position of the inferred binding pocket outlined in black. (a) Residues identified by Christinet *et al*. (2004) displayed on BvDODAα1. Tan residues show the substrate orienting (A, D, E) and ferrous cofactor orienting (B, C, F, G) residues. Residues are outlined in red if they contain a convergent substitution along any two focal branches, and grey if they contain a unique substitution along any branch. The residues identified by Christinet *et al*. (2004) as distinguishing Caryophyllales from non‐Caryophyllales DODA homologues, P‐(S,A)‐(N,D)‐x‐T‐P, are identified according to their states in BvDODAα1. (b, c) Residues identified by Bean *et al*. (2018) displayed on BvDODAα1. Labels gives the position in BvDODAα2 originally indexed by Bean *et al*. (2018), position in BvDODAα1, and the position in our alignment. Left view faces the binding pocket (a), and right view is rotated 180° (b). (d) Residues corresponding to sites with evidence for adaptive convergence. Labels give the corresponding alignment position. Outlined residue labels show sites supported by all four methods. (e–h) Convergent, divergent and unique substitutions inferred by ancestral sequence reconstruction. Predicted structures of four extant proteins from *Stegnosperma halimifolium* (ShDODAα1), *Beta vulgaris* (BvDODAα1), *Mesembryanthemum crystallinum* (McDODAα1), and *Carnegiea gigantea* (CgDODAα1). Sites inferred to experience substitutions along multibranch lineages ancestral to that protein are displayed. (i–l) The same four structures with substituting sites coloured according to their divergence between DODAα1‐like and DODAα2‐like homologs between each clade, and their conservation within each clade. Higher values show fewer overlapping states between clades and more conservation within clades. In each case, homologs of each organismal lineage are used (i.e. for BvDODAα1, Amaranthaceae DODAα1 homologs are compared to Amaranthaceae DODAα2 and DODAα4 homologs). (m–p) Kernel densities of distances of substituting sites from the predicted binding pocket centroid for all substitutions across branch/lineage for the same four structures. Red, convergent; blue, divergent; dark grey, unique; with the black line representing sites that do not experience any substitutions along our focal branch(es). DODA, l‐3,4‐dihydroxyphenylalanine (l‐DOPA) 4,5‐dioxygenase..

We first mapped important residues as originally determined by Christinet *et al*. (2004), and later by Bean *et al*. (2018) (Fig. 6a,b) to the structure of the BvDODAα1, together with the inferred binding pocket. For comparison, we then mapped the residues identified by at least three model‐based methods, visualised again on BvDODAα1 (Fig. 6c). To explore the potential functional significance of inferred substitutions from the ancestral sequence reconstruction analyses, we then mapped all changes inferred by comparing end states in the final reconstructed ancestor of each lineage (Stegnospermataceae, Amaranthaceae, Raphide and Portulacineeae) and mapped them to predicted structures of representative extant sequences from each major clade of DODAα1‐like sequences, alongside the predicted binding pocket (Fig. 6e–g). We calculated the Jensen‐Shannon Divergence between site‐specific amino acid frequencies for each DODAα1 clade and its sister DODAα2‐like sequences within each origin and calculated the conservation of amino acids for each site within each DODAα1 and DODAα2 clade, and combined these into a single metric such that high scoring sites were fixed on different states between DODAα1 and DODAα2 (Catania *et al*., 2024) (Fig. 6i–l). Note that since we only had a single pair of genes for Stegnospermataceae, divergence and conservation were drawn from the DODAα1 vs DODAα2 sequences over the whole alignment. Across all visualizations, we observed that inferred convergent substitutions tend to be clustered around the predicted binding pocket, but that many divergent substitutions are also proximal. To quantify this, we compared the distance to the binding pocket centroid of sites containing substitutions along our focal lineages to those with no substitutions inferred, measured from representative extant structures (Fig. 6m–p). In Stegnospermataceae, divergent substitutions, but not convergent or unique substitutions, tended to occur in sites closer to the binding pocket (one‐sided Wilcoxon rank‐sum with Holm correction, *P* = 0.0199, Fig. 6m). In Amaranthaceae, the density of convergent and divergent substitutions was shifted toward closer sites, but this was not significant (Fig. 6n). In the Raphides, both convergent and divergent substituting sites were significantly closer (*P* = 0.0006 and *P* = 0.0382, Fig. 6o). Finally, in the Portulacineae, convergent substitutions were significantly closer (*P* = 0.013, Fig. 6p). We observed a similar clustering around the binding pocket when dividing each lineage into its constituent branches (Fig. S8). The Stegnospermataceae origin (Fig. 6e) is again notable as our visualization emphasizes the relative frequency of divergent and unique substitutions, many of which are proximal to the binding pocket. Notably, two sites in the binding pocket of *Sh*DODAα1 experience convergent substitutions: one with the first branch in Amaranthaceae (9–10) that is replaced with divergent states along the second branch in Amaranthaceae (10–11), and a second with the Raphide branch (14–15) (Fig. S8). Most of the residues identified by Christinet *et al*. (2004) and Bean *et al*. (2018) are also detected by our analyses. However, all three of our approaches (ancestral reconstruction, model‐based methods, and extant conservation/divergence) consistently identify previously unrecognised residues close to the binding pocket. Our approaches also highlight a significant number of convergent and divergent sites, distal to the binding pocket. For ease of interrogating these results, we have made ChimeraX sessions with predicted structures coloured by substitution type available at https://github.com/NatJWalker‐Hale/DODA_convergence.

## Discussion

### Multiple origins of high l‐DOPA 4,5‐dioxygenase activity

Previously, we showed that high levels of l‐DOPA 4,5‐dioxygenase activity were polyphyletic over the DODAα clade of extradiol dioxygenases in Caryophyllales and suggested that this represented multiple transitions to high activity (Sheehan *et al*., 2020). However, we were unable to preclude a single transition to high activity followed by multiple losses. Here, we resolve this question by quantifying the activity of resurrected ancestral proteins. Our quantification of ancestral protein activity is sensitive to uncertainty in the estimation of ancestral sequence, but we factored in uncertainty by characterizing both the most probable sequence (MAP) and generating a second sequence replacing states at ambiguous sites (AltAll; Eick *et al*., 2016). In general, the AltAll is a relatively conservative assay of statistical uncertainty (Eick *et al*., 2016); however, regardless of whether we consider MAP or AltAll scenarios, our data convincingly demonstrate multiple transitions to high levels of DODA. Because of the central role of DODA in the betalain biosynthesis pathway, this finding supports our proposal that betalains evolved multiple times in Caryophyllales (Sheehan *et al*., 2020). In previous studies based on phylogenetic reconstructions using pigment data (Sheehan *et al*., 2020), we inferred four shifts to betalain pigmentation within Caryophyllales, in the Stegnospermataceae, Amaranthaceae, the Raphide clade, and the Portulacineae (Fig. 1). However, here, we find evidence for only three specializations to betalain pigmentation, with transitions to DODA inferred in Stegnospermataceae and Amaranthaceae and a single transition inferred at the base of the Globular Inclusion clade. Enzyme promiscuity can be an important factor in the evolution of new enzymatic activities (Copley, 2015). Here, the marginal level of activity along the backbone and in DODAα2‐like sequences (Fig. 2a; Sheehan *et al*., 2020; Guerrero‐Rubio *et al*., 2023) likely represents ancestral promiscuity, which, following gene duplications, was repeatedly selected for in these three origins.

### Patterns of high l‐DOPA 4,5‐dioxygenase activity within origins

Having established multiple separate origins of high l‐DOPA 4,5‐dioxygenase activity, we then examined the trajectories to high activity. Within these origins, the activity can vary several‐fold, and activity in extant proteins can also be lower than that of ancestral proteins, consistent with previous studies that have observed that reconstructed ancestral enzymes can show kinetic parameters that are more efficient than their extant descendants (e.g. Perez‐Jimenez *et al*., 2011). In most cases, the activities inferred from the MAP and AltAll were very similar, in line with Eick *et al*. (2016). However, uncertainty in ancestral activity at two nodes (9 and 13), highlighted by discrepancies between MAP and AltAll expression, allows for alternative scenarios of transitions to high DODA. For instance, at node 9, the MAP and AltAll estimates diverge regarding the ancestor of Amaranthaceae DODAα1 and DODAα4. In some cases, our MAP and AltAll expression seem to indicate a continuous increase of activity over multiple successive branches, perhaps implying specialization of DODA following initial transitions. However, we are reluctant to overinterpret quantitative differences in activity here, because we have not measured protein expression in our colonies and are effectively assuming equivalent protein concentrations, meaning that some quantitative differences could be due to differences in expression despite our codon optimization of expressed sequences for yeast (Van Gelder *et al*., 2024). In general, we do not think that this is likely to have a major impact on our results because measures of pigmentation in multiple heterologous systems match measurements of activity from purified enzymes (Guerrero‐Rubio *et al*., 2023). However, further demonstration of quantitative differences in activity from the reconstructed enzymes would require purification and comparative *in vitro* characterization, which presents considerable technical challenges. Prompted by these preliminary differences, for the purposes of subsequent analyses we considered substitutions over multiple branches to explore more inclusively the molecular basis of increasing activity within each origin.

### Mutational pathways to high l‐DOPA 4,5‐dioxygenase activity

Sheehan *et al*. (2020) conducted an analysis of seven sites identified as functionally important by Bean *et al*. (2018), and suggested molecular convergence had occurred in the DODA lineage. Here, we expanded these analyses to the entire protein using ancestral sequence reconstruction and leveraged our three well‐supported origins of high l‐DOPA 4,5‐dioxygenase activity. We reveal that a substantial number of amino acid substitutions occur along relevant branches within each of the three origins: 75 in Stegnospermataceae, 52 in Amaranthaceae, and 100 in the Globular Inclusion clade. We classified these substitutions into three categories: convergent (sharing the same amino acid identity across origins), divergent (occurring at the same position across origins but with different amino acids), and unique (occurring at novel positions, peculiar to individual origins). Each origin contained substitutions from all categories, though in varying proportions. Convergent residues, for example, made up only 8% of substitutions in the Stegnospermataceae origin but reached 35% in the Amaranthaceae origin. Divergent residues were consistently more frequent than convergent residues across all origins, from 44% in Amaranthaceae to 60% in the Globular Inclusion clade. As a combined class, convergent and divergent residues were significantly more prevalent than unique substitutions, comprising 58% in Stegnospermataceae, 79% in Amaranthaceae, and 83% in the Globular Inclusion clade. This overrepresentation highlights the frequent recurrence of similar amino acid substitutions across different origins, involving far more sites and substitutions than previously estimated (Christinet *et al*., 2004; Bean *et al*., 2018; Sheehan *et al*., 2020). But interestingly, the order of convergent and divergent substitutions varies among origins when comparing branches across evolutionary lineages. For instance, 50% of the substitutions occurring on the initial branch within Amaranthaceae are convergent or divergent with substitutions on later branches within the Globular Inclusion clade. This observation also supports our assumption that multiple successive evolutionary branches are involved in the evolution of high l‐DOPA 4,5‐dioxygenase. But remarkably, despite these recurring patterns, only two convergent residues are shared among all three origins, which may be due in part to the unique evolutionary trajectory toward high activity within Stegnospermataceae. The DODAα1 in the Stegnospermataceae origin is significantly displaced from other DODAα1‐like enzymes in sequence space and is enriched for unique substitutions and divergent states, even at highly conserved sites. Furthermore, our analysis does not account for variations due to insertions and deletions which also appear to have frequently occurred in *Sh*DODAα1 (Fig. S9), potentially facilitating its distinctive evolutionary path. These nuanced patterns underscore the complexity of evolutionary pathways to high activity across different lineages and origins. But in essence our ancestral sequence reconstruction reveals up to 62 substitutions with the same or similar amino acid identities and positions across the three origins, making the case for substantial molecular convergence.

### Evidence for adaptive convergence in high l‐DOPA 4,5‐dioxygenase activity

Despite the abundance of convergent and divergent residues detected by ancestral sequence reconstruction, there are legitimate concerns of false inference due to incidental neutral convergence, together with uncertainty associated with ancestral sequence reconstruction. We therefore employed additional methods to validate molecular convergence. In this context, two lines of evidence support adaptive convergence in the molecular evolution of DODA. First, our statistical tests and parametric bootstrapping with simulation showed that more convergence was observed among these different origins than was expected under ‘neutral’ expectations (Figs 4a,b, S7; Zou & Zhang, 2015), but also that the Stegnospermataceae origin is enriched for divergence and lacks significant convergence with the other origins. A variety of model‐based methods have been developed to detect adaptive convergence from aligned amino acid sequences (Tamuri *et al*., 2009; Chabrol *et al*., 2018; Parto & Lartillot, 2018; Rey *et al*., 2018, 2019). Here, our application of those approaches shows significant results. The different approaches infer between 7 and 62 substitutions, depending on approach, with many sites inferred by all or most methods. These sites overlap with those we infer to have experienced convergent and divergent substitutions based on ancestral sequence reconstruction. The former is unsurprising because the methods are eliciting similar statistical signals. The latter is also understandable, because the methods either do not consider amino acid identity explicitly (e.g. the branch‐site test) or consider an expanded definition of adaptive convergence where different amino acids can be similarly fit in the convergent environment, thus divergent substitutions can also represent adaptive convergence (Rey *et al*., 2019; Duchemin *et al*., 2023). That the consensus of the approaches is a much smaller subset of sites than implicated by ancestral sequence reconstruction likely reflects the signal of sites convergent in only a subset of lineages, together with neutral convergence due to drift (Rey *et al*., 2019). Thus, for example, the only site that is perfectly convergent between all three origins in our study (D564E) shows relatively low conservation within and divergence between DODAα1 and DODAα2, indicating that it may substitute frequently, and the observed convergence is due to chance. Despite the differences in these results, collectively they support the proposition that numerous substitutions in DODA have been driven by convergent adaptation, presumably for, but not necessarily exclusively for, increased l‐DOPA 4,5‐dioxygenase activity.

### Exploring convergence, divergence, and novelty in the context of protein structure

Previously, multiple authors have leveraged variation between extant DODAs in conjunction with structural information to implicate the residues that specify l‐DOPA 4,5‐dioxygenase activity. Christinet *et al*. (2004) identified multiple residues, including catalytic histidines widely conserved in extradiol dioxygenases, as potentially responsible for the orientation of aromatic substrates in the binding pocket. One of these residues is followed by a motif that differed between betalain and non‐betalain DODAs, which they indicated could potentially control access to the binding pocket (positions 650–655 in our alignment, Fig. 3). Bean *et al*. (2018) later described an additional set of sites that were different in extant DODAα1‐ and DODAα2‐like sequences (positions 543–547 in our alignment, Fig. 3) which might similarly control access to the active site (but occur on the opposite side to those highlighted by Christinet *et al*. (2004)). Recently, Chiang *et al*. (2025) used site‐directed mutagenesis to explore the role of several residues in l‐DOPA 4,5‐dioxygenase activity in *Bv*DODAα2. Despite DODAα2 being an isoform with only marginal activity, their results can still implicate key sites (Fig. 3). For example, their position 17 (position 475 in our alignment, Fig. 3) contains multiple convergent substitutions, and mutating from threonine to alanine in BvDODAα2 nearly abolished l‐DOPA 4,5‐dioxygenase activity, supporting our inference as to the functional importance of this position for the evolution of activity. All these prior studies capture only a subset of the substitutions identified in our analysis. Here, we mapped all substitutions emphasized by our ancestral reconstruction and convergence‐detection methods onto their respective protein structures (Fig. 6). These visualizations reveal that the majority of substitutions are clustered near the binding pocket. Notably, divergent substitutions frequently occur near the binding pocket across all origins, highlighting the possible functional significance of these residue changes despite the lack of conserved amino acid identities across origins. This pattern is particularly pronounced in the Stegnospermataceae origin, where only a single convergent substitution, but many divergent and unique substitutions, are found in the binding pocket. This underscores the distinctive molecular evolutionary pathway in this lineage. The exact structural and enzymatic impact of these lineage‐specific novelties remains to be explored, but one prediction might be that these lineage‐specific divergences cannot generalize to sequence backgrounds from other transitions. An alternative possibility is that different amino‐acid states are similarly fit in the convergent environment, in which case they should have similar effects regardless of lineage‐specific sequence background. Beyond the binding pocket, a notable number of convergent, divergent, and unique substitutions also appear in corresponding structural regions distal to the active site. There is well‐established and increasing evidence for the functional importance of such distal mutations, including for structural stability and flexibility, allosteric regulation, substrate channelling or binding orientation, protein interaction, or adaptation to cellular environments (Yang *et al*., 2020; Osuna, 2021). Testing these hypotheses, including the combinatorial effect of proximal and distal mutations, will require large‐scale directed mutagenesis to further explore the mutational and phenotypic landscape.

### Conclusion

We have demonstrated that high l‐DOPA 4,5‐dioxygenase activity in Caryophyllales evolved independently three times from low‐activity ancestral forms. Since l‐DOPA 4,5‐dioxygenase is essential for betalain biosynthesis, this finding strongly supports accumulating evidence for the convergent origins of betalains within Caryophyllales (Sheehan *et al*., 2020; Pucker *et al*., 2024). Harnessing the phenomenon of convergence, we gained additional insights into the molecular evolution of high activity. Our findings support the key role of gene duplication in generating biochemical novelty in plants, as each of our inferred transitions occurs following an independent gene duplication. Because our low activity ancestral sequences almost always show some level of activity, consistent with extant sequences, this supports a model already explored in the context of anthocyanin pigmentation, where selection on a promiscuous activity is enabled by gene duplication (Des Marais & Rausher, 2008). Unlike earlier studies that have suggested a small number of large‐effect residues (Christinet *et al*., 2004; Bean *et al*., 2018), our findings align more with Guerrero‐Rubio *et al*. (2023), suggesting a more complex scenario, with up to 64 overlapping substitutions arising across the three origins. Our statistical analysis provides strong evidence of adaptive molecular convergence, with substitutions clustering around the active site, suggesting that sequences have converged on similar genotypes during transitions to high activity. But equally compelling evidence for divergence and novelty emerges both among and within these origins, which manifests in divergent and unique substitutions close to the binding pocket, as well as the highly divergent sequence of DODAα1 in Stegnospermataceae. The varied trajectory of this latter sequence could be a product of contingency (Xie *et al*., 2021), and the states involved in specific divergent substitutions in Stegnosperma and other lineages might be the product of epistasis with lineage‐specific sequence backgrounds (Starr & Thornton, 2016). Other plant pigmentation enzymes and pathways have shown evidence for epistasis during switches in substrate specificity. For example, Dihydroflavanol Reductase showed negative epistasis among mutations controlling substrate specificity on different dihydroflavonol substrates during a flower colour transition (Smith *et al*., 2013), and positive epistasis was recently implicated in the formation of specificity for salicylic acid in a family of plant methyltransferases (Catania *et al*., 2024). Our convergence‐based approaches in conjunction with structural modelling also highlight the potential importance of substitutions distal to the active site, indicating potential novel and undiscovered aspects of DODA enzyme structure and function. Given the dimensions of the combinatorial substitution space, we are yet to functionally validate these results, but our findings underscore the complex molecular pathways underlying the evolution of DODA function. Disentangling the complexity of this molecular evolution will be a significant challenge. However, the betalain pathway is well‐suited as a high throughput experimental system for molecular evolution of enzyme function, given its viability across multiple microbial platforms, and the visible and fluorescent properties of betalain outputs.

## Competing interests

None declared.

## Author contributions

NW‐H, MAGR, and SFB planned and designed the research. NW‐H and MAGR performed experiments and analysed data. NW‐H and SFB prepared the figures. NW‐H, MAGR, and SFB wrote the manuscript. NW‐H and MAGR contributed equally.

## Disclaimer

The New Phytologist Foundation remains neutral with regard to jurisdictional claims in maps and in any institutional affiliations.

## Supporting information


**Fig. S1** Species tree used for maximum likelihood reconciliation.
**Fig. S2** Maximum likelihood reconciliation of DODAα sequences from GeneRax.
**Fig. S3** Modified reconciled topology.
**Fig. S4** Final pruned reconciled topology.
**Fig. S5** A paralogue of Spinach DODAα1 shows low activity.
**Fig. S6** Posterior probabilities of reconstructed sequences.
**Fig. S7** Expected convergence and divergence based on parametric bootstrapping with simulation.
**Fig. S8** Structural context of inferred substitutions per branch.
**Fig. S9** Comparison of states in ShDODAα1, ShDODAα2, and inferred ancestors.
**Table S1** Information on primers employed in this work.
**Table S2** Information on *Saccharomyces cerevisiae* strains constructed for this work.
Please note: Wiley is not responsible for the content or functionality of any Supporting Information supplied by the authors. Any queries (other than missing material) should be directed to the *New Phytologist* Central Office.

## Data Availability

Codon‐optimized ancestral reconstructions and extant sequences are available at the NCBI under accessions PV487753‐PV487786 and PV476478‐PV476524, respectively. All alignments, trees, inferred sequences, expression data, and predicted structures underlying this work are available from: https://github.com/NatJWalker‐Hale/DODA_convergence and archived on Zenodo: https://doi.org/10.5281/zenodo.15185291.

## References

[nph70177-bib-0001] Bean A , Sunnadeniya R , Akhavan N , Campbell A , Brown M , Lloyd A . 2018. Gain‐of‐function mutations in beet DODA2 identify key residues for betalain pigment evolution. New Phytologist 219: 287–296. 29754447 10.1111/nph.15159

[nph70177-bib-0002] Besnard G , Muasya AM , Russier F , Roalson EH , Salamin N , Christin P‐A . 2009. Phylogenomics of C_4_ photosynthesis in sedges (Cyperaceae): multiple appearances and genetic convergence. Molecular Biology and Evolution 26: 1909–1919. 19461115 10.1093/molbev/msp103

[nph70177-bib-0003] Brockington SF , Walker RH , Glover BJ , Soltis PS , Soltis DE . 2011. Complex pigment evolution in the Caryophyllales. New Phytologist 190: 854–864. 21714182 10.1111/j.1469-8137.2011.03687.x

[nph70177-bib-0004] Brockington SF , Yang Y , Gandia‐Herrero F , Covshoff S , Hibberd JM , Sage RF , Wong GKS , Moore MJ , Smith SA . 2015. Lineage‐specific gene radiations underlie the evolution of novel betalain pigmentation in Caryophyllales. New Phytologist 207: 1170–1180. 25966996 10.1111/nph.13441 PMC4557044

[nph70177-bib-0005] Brown JW , Walker JF , Smith SA . 2017. Phyx: phylogenetic tools for unix. Bioinformatics 33: 1886–1888. 28174903 10.1093/bioinformatics/btx063 PMC5870855

[nph70177-bib-0006] Castoe TA , Koning APJ , Kim H‐M , Gu W , Noonan BP , Naylor G , Jiang ZJ , Parkinson CL , Pollock DD . 2009. Evidence for an ancient adaptive episode of convergent molecular evolution. Proceedings of the National Academy of Sciences, USA 106: 8986–8991. 10.1073/pnas.0900233106 PMC2690048 19416880

[nph70177-bib-0007] Catania EM , Dubs NM , Soumen S , Barkman TJ . 2024. The mutational road not taken: using ancestral sequence resurrection to evaluate the evolution of plant enzyme substrate preferences. Genome Biology and Evolution 16: evae016. 38290535 10.1093/gbe/evae016 PMC10853004

[nph70177-bib-0008] Chabrol O , Royer‐Carenzi M , Pontarotti P , Didier G . 2018. Detecting the molecular basis of phenotypic convergence. Methods in Ecology and Evolution 9: 2170–2180.

[nph70177-bib-0081] Chiang C‐C , Lu Y‐J , Liu J‐W , Lin S‐W , Chou C‐C , Lin C‐H , Chien I‐W , Hsu C‐H . 2025. Structural insights into 4,5‐DOPA extradiol dioxygenase from *Beta vulgaris*: unraveling the key step in versatile betalain biosynthesis. Journal of Agricultural and Food Chemistry 73(11): 6785–6794. 40055856 10.1021/acs.jafc.4c09501 PMC11926856

[nph70177-bib-0009] Christin P‐A , Salamin N , Savolainen V , Duvall MR , Besnard G . 2007. C_4_ photosynthesis evolved in grasses via parallel adaptive genetic changes. Current Biology 17: 1241–1247. 17614282 10.1016/j.cub.2007.06.036

[nph70177-bib-0010] Christin P‐A , Weinreich DM , Besnard G . 2010. Causes and evolutionary significance of genetic convergence. Trends in Genetics 26: 400–405. 20685006 10.1016/j.tig.2010.06.005

[nph70177-bib-0011] Christinet L , Burdet FX , Zaiko M , Hinz U , Zrÿd J‐P . 2004. Characterization and functional identification of a novel plant 4,5‐extradiol dioxygenase involved in betalain pigment biosynthesis in *Portulaca grandiflora* . Plant Physiology 134: 265–274. 14730069 10.1104/pp.103.031914 PMC316306

[nph70177-bib-0012] Chung H‐H , Schwinn KE , Ngo HM , Lewis DH , Massey B , Calcott KE , Crowhurst R , Joyce DC , Gould KS , Davies KM *et al*. 2015. Characterisation of betalain biosynthesis in Parakeelya flowers identifies the key biosynthetic gene DOD as belonging to an expanded LigB gene family that is conserved in betalain‐producing species. Frontiers in Plant Science 6. 10.3389/fpls.2015.00499 PMC4493658 26217353

[nph70177-bib-0013] Copley SD . 2015. An evolutionary biochemist's perspective on promiscuity. Trends in Biochemical Sciences 40: 72–78. 25573004 10.1016/j.tibs.2014.12.004 PMC4836852

[nph70177-bib-0014] Davydov II , Salamin N , Robinson‐Rechavi M . 2019. Large‐scale comparative analysis of codon models accounting for protein and nucleotide selection. Molecular Biology and Evolution 36: 1316–1332. 30847475 10.1093/molbev/msz048 PMC6526913

[nph70177-bib-0015] Des Marais DL , Rausher MD . 2008. Escape from adaptive conflict after duplication in an anthocyanin pathway gene. Nature 454: 762–765. 18594508 10.1038/nature07092

[nph70177-bib-0016] Duchemin L , Lanore V , Veber P , Boussau B . 2023. Evaluation of methods to detect shifts in directional selection at the genome scale. Molecular Biology and Evolution 40: msac247. 36510704 10.1093/molbev/msac247 PMC9940701

[nph70177-bib-0017] Eick GN , Bridgham JT , Anderson DP , Harms MJ , Thornton JW . 2016. Robustness of reconstructed ancestral protein functions to statistical uncertainty. Molecular Biology and Evolution 34: 247–261. 10.1093/molbev/msw223 PMC6095102 27795231

[nph70177-bib-0018] Foote AD , Liu Y , Thomas GWC , Vinař T , Alföldi J , Deng J , Dugan S , van Elk CE , Hunter ME , Joshi V *et al*. 2015. Convergent evolution of the genomes of marine mammals. Nature Genetics 47: 272–275. 25621460 10.1038/ng.3198 PMC4644735

[nph70177-bib-0019] Gietz RD , Schiestl RH . 2007. High‐efficiency yeast transformation using the LiAc/SS carrier DNA/PEG method. Nature Protocols 2: 31–34. 17401334 10.1038/nprot.2007.13

[nph70177-bib-0021] Guerrero‐Rubio MA , Walker‐Hale N , Guo R , Sheehan H , Timoneda A , Gandia‐Herrero F , Brockington SF . 2023. Are seven amino acid substitutions sufficient to explain the evolution of high l‐DOPA 4,5‐dioxygenase activity leading to betalain pigmentation? Revisiting the gain‐of‐function mutants of Bean et al. (2018). New Phytologist 239: 2265–2276. 37243529 10.1111/nph.18981

[nph70177-bib-0022] Heyduk K , Moreno‐Villena JJ , Gilman IS , Christin P‐A , Edwards EJ . 2019. The genetics of convergent evolution: insights from plant photosynthesis. Nature Reviews. Genetics 20: 485–493. 10.1038/s41576-019-0107-5 30886351

[nph70177-bib-0023] Hochberg GKA , Thornton JW . 2017. Reconstructing ancient proteins to understand the causes of structure and function. Annual Review of Biophysics 46: 247–269. 10.1146/annurev-biophys-070816-033631 PMC6141191 28301769

[nph70177-bib-0024] Hoekstra HE , Hirschmann RJ , Bundey RA , Insel PA , Crossland JP . 2006. A single amino acid mutation contributes to adaptive beach mouse color pattern. Science 313: 101–104. 16825572 10.1126/science.1126121

[nph70177-bib-0026] Huang R , O'Donnell AJ , Barboline JJ , Barkman TJ . 2016. Convergent evolution of caffeine in plants by co‐option of exapted ancestral enzymes. Proceedings of the National Academy of Sciences, USA 113: 10613–10618. 10.1073/pnas.1602575113 PMC5035902 27638206

[nph70177-bib-0029] Jumper J , Evans R , Pritzel A , Green T , Figurnov M , Ronneberger O , Tunyasuvunakool K , Bates R , Žídek A , Potapenko A *et al*. 2021. Highly accurate protein structure prediction with AlphaFold. Nature 596: 583–589. 34265844 10.1038/s41586-021-03819-2 PMC8371605

[nph70177-bib-0030] Kaltenbach M , Burke JR , Dindo M , Pabis A , Munsberg FS , Rabin A , Kamerlin SCL , Noel JP , Tawfik DS . 2018. Evolution of chalcone isomerase from a noncatalytic ancestor. Nature Chemical Biology 14: 548–555. 29686356 10.1038/s41589-018-0042-3

[nph70177-bib-0031] Kasei A , Watanabe H , Ishiduka N , Noda K , Murata M , Sakuta M . 2021. Comparative analysis of the extradiol ring‐cleavage dioxygenase ligb from arabidopsis and 3,4‐dihydroxyphenylalanine dioxygenase from betalain‐producing plants. Plant and Cell Physiology 62: 732–740. 33638982 10.1093/pcp/pcab031

[nph70177-bib-0032] Katoh K , Standley DM . 2013. MAFFT multiple sequence alignment software version 7: improvements in performance and usability. Molecular Biology and Evolution 30: 772–780. 23329690 10.1093/molbev/mst010 PMC3603318

[nph70177-bib-0033] Kozlov AM , Darriba D , Flouri T , Morel B , Stamatakis A . 2019. RAxML‐NG: a fast, scalable and user‐friendly tool for maximum likelihood phylogenetic inference. Bioinformatics 35: 4453–4455. 31070718 10.1093/bioinformatics/btz305 PMC6821337

[nph70177-bib-0034] Krivák R , Hoksza D . 2018. P2Rank: machine learning based tool for rapid and accurate prediction of ligand binding sites from protein structure. Journal of Cheminformatics 10: 39. 30109435 10.1186/s13321-018-0285-8 PMC6091426

[nph70177-bib-0035] Lee ME , DeLoache WC , Cervantes B , Dueber JE . 2015. A highly characterized yeast toolkit for modular, multipart assembly. ACS Synthetic Biology 4: 975–986. 25871405 10.1021/sb500366v

[nph70177-bib-0036] Lichman BR , Godden GT , Hamilton JP , Palmer L , Kamileen MO , Zhao D , Vaillancourt B , Wood JC , Sun M , Kinser TJ *et al*. 2020. The evolutionary origins of the cat attractant nepetalactone in catnip. Science Advances 6: eaba0721. 32426505 10.1126/sciadv.aba0721 PMC7220310

[nph70177-bib-0037] Losos JB . 2011. Convergence, adaptation, and constraint. Evolution 65: 1827–1840. 21729041 10.1111/j.1558-5646.2011.01289.x

[nph70177-bib-0038] Löytynoja A , Goldman N . 2005. An algorithm for progressive multiple alignment of sequences with insertions. Proceedings of National Academy of Sciences, USA 102: 10557–10562. 10.1073/pnas.0409137102 PMC1180752 16000407

[nph70177-bib-0039] Meng EC , Goddard TD , Pettersen EF , Couch GS , Pearson ZJ , Morris JH , Ferrin TE . 2023. UCSF ChimeraX: tools for structure building and analysis. Protein Science 32: e4792. 37774136 10.1002/pro.4792 PMC10588335

[nph70177-bib-0040] Minh BQ , Schmidt HA , Chernomor O , Schrempf D , Woodhams MD , von Haeseler A , Lanfear R . 2020. IQ‐TREE 2: new models and efficient methods for phylogenetic inference in the genomic era. Molecular Biology and Evolution 37: 1530–1534. 32011700 10.1093/molbev/msaa015 PMC7182206

[nph70177-bib-0041] Mirdita M , Schütze K , Moriwaki Y , Heo L , Ovchinnikov S , Steinegger M . 2022. ColabFold: making protein folding accessible to all. Nature Methods 19: 679–682. 35637307 10.1038/s41592-022-01488-1 PMC9184281

[nph70177-bib-0042] Moghe GD , Last RL . 2015. Something old, something new: conserved enzymes and the evolution of novelty in plant specialized metabolism. Plant Physiology 169: 1512–1523. 26276843 10.1104/pp.15.00994 PMC4634076

[nph70177-bib-0043] Morel B , Kozlov AM , Stamatakis A , Szöllősi GJ . 2020. GeneRax: a tool for species‐tree‐aware maximum likelihood‐based gene family tree inference under gene duplication, transfer, and loss. Molecular Biology and Evolution 37: 2763–2774. 32502238 10.1093/molbev/msaa141 PMC8312565

[nph70177-bib-0044] Natarajan C , Hoffmann FG , Weber RE , Fago A , Witt CC , Storz JF . 2016. Predictable convergence in hemoglobin function has unpredictable molecular underpinnings. Science 354: 336–339. 27846568 10.1126/science.aaf9070 PMC5464326

[nph70177-bib-0045] O'Donnell AJ , Huang R , Barboline JJ , Barkman TJ . 2021. Convergent biochemical pathways for xanthine alkaloid production in plants evolved from ancestral enzymes with different catalytic properties. Molecular Biology and Evolution 38: 2704–2714. 33662138 10.1093/molbev/msab059 PMC8233510

[nph70177-bib-0046] Osuna S . 2021. The challenge of predicting distal active site mutations in computational enzyme design. WIREs Computational Molecular Science 11: e1502.

[nph70177-bib-0047] Parto S , Lartillot N . 2017. Detecting consistent patterns of directional adaptation using differential selection codon models. BMC Evolutionary Biology 17: 147. 28645318 10.1186/s12862-017-0979-y PMC5481935

[nph70177-bib-0048] Parto S , Lartillot N . 2018. Molecular adaptation in Rubisco: discriminating between convergent evolution and positive selection using mechanistic and classical codon models. PLoS ONE 13: e0192697. 29432438 10.1371/journal.pone.0192697 PMC5809049

[nph70177-bib-0049] Perez‐Jimenez R , Inglés‐Prieto A , Zhao Z‐M , Sanchez‐Romero I , Alegre‐Cebollada J , Kosuri P , Garcia‐Manyes S , Kappock TJ , Tanokura M , Holmgren A *et al*. 2011. Single‐molecule paleoenzymology probes the chemistry of resurrected enzymes. Nature Structural & Molecular Biology 18: 592–596. 10.1038/nsmb.2020 PMC3087858 21460845

[nph70177-bib-0050] Pichersky E , Lewinsohn E . 2011. Convergent evolution in plant specialized metabolism. Annual Review of Plant Biology 62: 549–566. 10.1146/annurev-arplant-042110-103814 21275647

[nph70177-bib-0051] Pollock DD , Thiltgen G , Goldstein RA . 2012. Amino acid coevolution induces an evolutionary Stokes shift. Proceedings of the National Academy of Sciences, USA 109: E1352–E1359. 10.1073/pnas.1120084109 PMC3361410 22547823

[nph70177-bib-0082] Pucker B , Walker‐Hale N , Dzurlic J , Yim WC , Cushman JC , Crum A , Yang Y , Brockington SF . 2023. Multiple mechanisms explain loss of anthocyanins from betalain‐pigmented Caryophyllales, including repeated wholesale loss of a key anthocyanidin synthesis enzyme. New Phytologist 241(1): 471–489. 37897060 10.1111/nph.19341 PMC10952170

[nph70177-bib-0052] Pupko T , Pe I , Shamir R , Graur D . 2000. A fast algorithm for joint reconstruction of ancestral amino acid sequences. Molecular Biology and Evolution 17: 890–896. 10833195 10.1093/oxfordjournals.molbev.a026369

[nph70177-bib-0053] Pupko T , Pe'er I , Hasegawa M , Graur D , Friedman N . 2002. A branch‐and‐bound algorithm for the inference of ancestral amino‐acid sequences when the replacement rate varies among sites: application to the evolution of five gene families. Bioinformatics 18: 1116–1123. 12176835 10.1093/bioinformatics/18.8.1116

[nph70177-bib-0054] Rey C , Guéguen L , Sémon M , Boussau B . 2018. Accurate detection of convergent amino‐acid evolution with PCOC. Molecular Biology and Evolution 35: 2296–2306. 29986048 10.1093/molbev/msy114 PMC6106957

[nph70177-bib-0055] Rey C , Lanore V , Veber P , Guéguen L , Lartillot N , Sémon M , Boussau B . 2019. Detecting adaptive convergent amino acid evolution. Philosophical Transactions of the Royal Society, B: Biological Sciences 374: 20180234. 10.1098/rstb.2018.0234 PMC6560273 31154974

[nph70177-bib-0056] Sackton TB , Grayson P , Cloutier A , Hu Z , Liu JS , Wheeler NE , Gardner PP , Clarke JA , Baker AJ , Clamp M *et al*. 2019. Convergent regulatory evolution and loss of flight in paleognathous birds. Science 364: 74–78. 30948549 10.1126/science.aat7244

[nph70177-bib-0057] Sasaki N , Abe Y , Goda Y , Adachi T , Kasahara K , Ozeki Y . 2009. Detection of DOPA 4,5‐dioxygenase (DOD) activity using recombinant protein prepared from escherichia coli cells harboring cDNA encoding DOD from *Mirabilis jalapa* . Plant & Cell Physiology 50: 1012–1016. 19366710 10.1093/pcp/pcp053

[nph70177-bib-0058] Sheehan H , Feng T , Walker‐Hale N , Lopez‐Nieves S , Pucker B , Guo R , Yim WC , Badgami R , Timoneda A , Zhao L *et al*. 2020. Evolution of l‐DOPA 4,5‐dioxygenase activity allows for recurrent specialisation to betalain pigmentation in Caryophyllales. New Phytologist 227: 914–929. 31369159 10.1111/nph.16089 PMC7384185

[nph70177-bib-0059] Smith SA , Walker JF . 2019. PyPHLAWD: a python tool for phylogenetic dataset construction. Methods in Ecology and Evolution 10: 104–108.

[nph70177-bib-0060] Smith SD , Wang S , Rausher MD . 2013. Functional evolution of an anthocyanin pathway enzyme during a flower color transition. Molecular Biology and Evolution 30: 602–612. 23155005 10.1093/molbev/mss255 PMC3563968

[nph70177-bib-0061] Starr TN , Thornton JW . 2016. Epistasis in protein evolution. Protein Science 25: 1204–1218. 26833806 10.1002/pro.2897 PMC4918427

[nph70177-bib-0062] Stayton CT . 2008. Is convergence surprising? An examination of the frequency of convergence in simulated datasets. Journal of Theoretical Biology 252: 1–14. 18321532 10.1016/j.jtbi.2008.01.008

[nph70177-bib-0063] Stayton CT . 2015. What does convergent evolution mean? The interpretation of convergence and its implications in the search for limits to evolution. Interface Focus 5: 20150039. 26640646 10.1098/rsfs.2015.0039 PMC4633856

[nph70177-bib-0064] Stern DL . 2013. The genetic causes of convergent evolution. Nature Reviews. Genetics 14: 751–764. 10.1038/nrg3483 24105273

[nph70177-bib-0065] Storz JF . 2016. Causes of molecular convergence and parallelism in protein evolution. Nature Reviews. Genetics 17: 239–250. 10.1038/nrg.2016.11 PMC5482790 26972590

[nph70177-bib-0066] Sunnadeniya R , Bean A , Brown M , Akhavan N , Hatlestad G , Gonzalez A , Symonds VV , Lloyd A . 2016. Tyrosine hydroxylation in betalain pigment biosynthesis is performed by cytochrome P450 enzymes in beets (*Beta vulgaris*). PLoS ONE 11: e0149417. 26890886 10.1371/journal.pone.0149417 PMC4758722

[nph70177-bib-0067] Tamuri AU , dos Reis M , Hay AJ , Goldstein RA . 2009. Identifying changes in selective constraints: host shifts in influenza. PLoS Computational Biology 5: e1000564. 19911053 10.1371/journal.pcbi.1000564 PMC2770840

[nph70177-bib-0068] Timoneda A , Feng T , Sheehan H , Walker‐Hale N , Pucker B , Lopez‐Nieves S , Guo R , Brockington S . 2019. The evolution of betalain biosynthesis in Caryophyllales. New Phytologist 224: 71–85. 31172524 10.1111/nph.15980

[nph70177-bib-0069] Van Belleghem SM , Ruggieri AA , Concha C , Livraghi L , Hebberecht L , Rivera ES , Ogilvie JG , Hanly JJ , Warren IA , Planas S *et al*. 2023. High level of novelty under the hood of convergent evolution. Science 379: 1043–1049. 36893249 10.1126/science.ade0004 PMC11000492

[nph70177-bib-0070] Van Gelder K , Lindner SN , Hanson AD , Zhou J . 2024. Strangers in a foreign land: ‘Yeastizing’ plant enzymes. Microbial Biotechnology 17: e14525. 39222378 10.1111/1751-7915.14525 PMC11368087

[nph70177-bib-0071] Walker JF , Yang Y , Feng T , Timoneda A , Mikenas J , Hutchison V , Edwards C , Wang N , Ahluwalia S , Olivieri J *et al*. 2018. From cacti to carnivores: improved phylotranscriptomic sampling and hierarchical homology inference provide further insight into the evolution of Caryophyllales. American Journal of Botany 105: 446–462. 29738076 10.1002/ajb2.1069

[nph70177-bib-0072] Weng J‐K , Li Y , Mo H , Chapple C . 2012. Assembly of an evolutionarily new pathway for α‐pyrone biosynthesis in Arabidopsis. Science 337: 960–964. 22923580 10.1126/science.1221614

[nph70177-bib-0073] Xie VC , Pu J , Metzger BP , Thornton JW , Dickinson BC . 2021. Contingency and chance erase necessity in the experimental evolution of ancestral proteins. eLife 10: e67336. 34061027 10.7554/eLife.67336 PMC8282340

[nph70177-bib-0074] Yang G , Miton CM , Tokuriki N . 2020. A mechanistic view of enzyme evolution. Protein Science 29: 1724–1747. 32557882 10.1002/pro.3901 PMC7380680

[nph70177-bib-0075] Yang Y , Smith SA . 2014. Orthology inference in nonmodel organisms using transcriptomes and low‐coverage genomes: improving accuracy and matrix occupancy for phylogenomics. Molecular Biology and Evolution 31: 3081–3092. 25158799 10.1093/molbev/msu245 PMC4209138

[nph70177-bib-0076] Yang Z . 1997. PAML: a program package for phylogenetic analysis by maximum likelihood. Bioinformatics 13: 555–556. 10.1093/bioinformatics/13.5.555 9367129

[nph70177-bib-0077] Yang Z . 2014. Molecular evolution: a statistical approach. Oxford, UK: Oxford University Press.

[nph70177-bib-0078] Zhang J , Kumar S . 1997. Detection of convergent and parallel evolution at the amino acid sequence level. Molecular Biology and Evolution 14: 527–536. 9159930 10.1093/oxfordjournals.molbev.a025789

[nph70177-bib-0079] Zhang J , Nielsen R , Yang Z . 2005. Evaluation of an improved branch‐site likelihood method for detecting positive selection at the molecular level. Molecular Biology and Evolution 22: 2472–2479. 16107592 10.1093/molbev/msi237

[nph70177-bib-0080] Zou Z , Zhang J . 2015. Are convergent and parallel amino acid substitutions in protein evolution more prevalent than neutral expectations? Molecular Biology and Evolution 32: 2085–2096. 25862140 10.1093/molbev/msv091 PMC4833076

