# Supplementary material for: Multiple transitions to high l‐DOPA 4,5‐dioxygenase activity reveal molecular pathways to convergent betalain pigmentation in Caryophyllales
Source: New Phytol. 2025 May 5;247(1):341–57. doi: 10.1111/nph.70177 (PMC12138177; doi:10.1111/nph.70177)
Supplement: Supplementary file 1 — Fig. S1 Species tree used for maximum likelihood reconciliation. Fig. S2 Maximum likelihood reconciliation of DODAα sequences from GeneRax. Fig. S3 Modified reconciled topology. Fig. S4 Final pruned reconciled topology. Fig. S5 A paralogue of Spinach DODAα1 shows low activity. Fig. S6 Posterior probabilities of reconstructed sequences. Fig. S7 Expected convergence and divergence based on parametric bootstrapping with simulation. Fig. S8 Structural context of inferred substitutions per branch. Fig. S9 Comparison of states in ShDODAα1, ShDODAα2, and inferred ancestors. Table S1 Information on primers employed in this work. Table S2 Information on Saccharomyces cerevisiae strains constructed for this work. Please note: Wiley is not responsible for the content or functionality of any Supporting Information supplied by the authors. Any queries (other than missing material) should be directed to the New Phytologist Central Office. [file NPH-247-341-s001.pdf]

***New Phytologist* Supporting Information**

Article title: Multiple transitions to high *L*-DOPA 4,5-dioxygenase activity reveal molecular pathways to convergent betalain pigmentation in Caryophyllales.

Authors: Nathanael Walker-Hale, M. Alejandra Guerrero-Rubio, Samuel F. Brockington

Article acceptance date: 4 April 2025

Figure S1: Species tree used for maximum likelihood reconciliation. Branch lengths are arbitrary. Tree was rooted on the clade of non-core Caryophyllales.

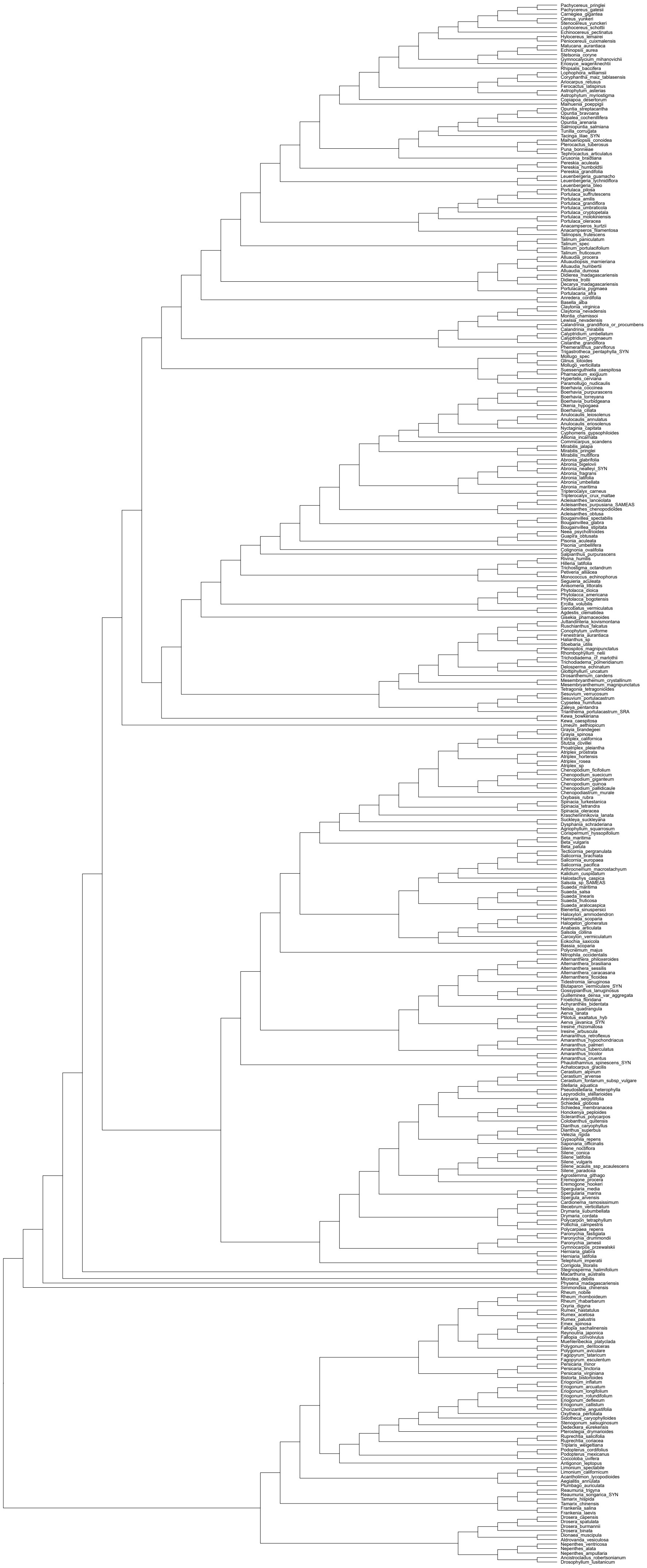

Pachycereus_pringlei
Pachycereus_gatesii
Carnegiea_gigantea
Cereus_yunkeri
Stenocereus_yunckeri
Lophocereus_schottii
Echinocereus_pectinatus
Hylocereus_lemairei
Peniocereus_cuixmalensis
Matucana_aurantiaca
Echinopsis_aurea
Stetsonia_coryne
Gymnocalycium_mihanovichii
Eriosyce_wagenknechtii
Rhipsalis_baccifera
Lophophora_williamsii
Coryphantha_maiz_tablasensis
Ariocarpus_retusus
Ferocactus_latispinus
Astrophytum_asterias
Astrophytum_myriostigma
Copiapoa_desertorum
Maihuenia_poeppigii
Opuntia_streptacantha
Opuntia_bravoana
Nopalea_cochenillifera
Opuntia_arenaria
Salmiopuntia_salmiana
Tunilla_corrugata
Tacinga_lilae_SYN
Maihueniopsis_conoidea
Pterocactus_tuberosus
Puna_bonnieae
Tephrocactus_articulatus
Grusonia_bradtiana
Pereskia_aculeata
Pereskia_humboldtii
Pereskia_grandifolia
Leuenbergeria_guamacho
Leuenbergeria_lychnidiflora
Leuenbergeria_bleo
Portulaca_pilosa
Portulaca_suffrutescens
Portulaca_amilis
Portulaca_grandiflora
Portulaca_umbraticola
Portulaca_cryptopetala
Portulaca_molokiniensis
Portulaca_oleracea
Anacampseros_kurtzii
Anacampseros_filamentosa
Talinopsis_frutescens
Talinum_paniculatum
Talinum_spec
Talinum_portulacifolium
Talinum_fruticosum
Alluaudia_procera
Alluaudiopsis_marnieriana
Alluaudia_humbertii
Alluaudia_dumosa
Didierea_madagascariensis
Didierea_trollii
Decarya_madagascariensis
Portulacaria_pygmaea
Portulacaria_afra
Anredera_cordifolia
Basella_alba
Claytonia_virginica
Claytonia_nevadensis
Montia_chamissoi
Lewisia_nevadensis
Calandrinia_grandiflora_or_procumbens
Calandrinia_mirabilis
Calyptridium_umbellatum
Calyptridium_pygmaeum
Cistanthe_grandiflora
Phemeranthus_parviflorus
Trigastrotheca_pentaphylla_SYN
Mollugo_spec
Glinus_lotoides
Mollugo_verticillata
Suessenguthiella_caespitosa
Pharnaceum_exiguum
Hypertelis_cerviana
Paramollugo_nudicaulis
Boerhavia_coccinea
Boerhavia_purpurascens
Boerhavia_torreyana
Boerhavia_burbidgeana
Okenia_hypogaea
Boerhavia_ciliata
Anulocaulis_leiosolenus
Anulocaulis_annulatus
Anulocaulis_eriosolenus
Nyctaginia_capitata
Cyphomeris_gypsophiloides
Allionia_incarnata
Commicarpus_scandens
Mirabilis_jalapa
Mirabilis_pringlei
Mirabilis_multiflora
Abronia_glabrifolia
Abronia_bigelovii
Abronia_nealleyi_SYN
Abronia_fragrans
Abronia_latifolia
Abronia_umbellata
Abronia_maritima
Tripterocalyx_carneus
Tripterocalyx_crux_maltae
Acleisanthes_lanceolata
Acleisanthes_purpusiana_SAMEAS
Acleisanthes_chenopodioides
Acleisanthes_obtusa
Bougainvillea_spectabilis
Bougainvillea_glabra
Bougainvillea_stipitata
Neea_psychotrioides
Guapira_obtusata
Pisonia_aculeata
Pisonia_umbellifera
Colignonia_ovalifolia
Salpianthus_purpurascens
Rivina_humilis
Hilleria_latifolia
Trichostigma_octandrum
Petiveria_alliacea
Monococcus_echinophorus
Seguieria_aculeata
Anisomeria_littoralis
Phytolacca_dioica
Phytolacca_americana
Phytolacca_bogotensis
Ercilla_volubilis
Sarcobatus_vermiculatus
Agdestis_clematidea
Gisekia_pharnaceoides
Juttandinteria_kovismontana
Ruschianthus_falcatus
Conophytum_uviforme
Fenestraria_aurantiaca
Halianthus_sp
Stoebaria_utilis
Pleiospilos_magnipunctatus
Rhombophyllum_nelii
Trichodiadema_cf_marlothii
Trichodiadema_pomeridianum
Delosperma_echinatum
Glottiphyllum_uncatum
Drosanthemum_candens
Mesembryanthemum_crystallinum
Mesembryanthemum_magnipunctatus
Tetragonia_tetragonioides
Sesuvium_verrucosum
Sesuvium_portulacastrum
Cypselea_humifusa
Zaleya_pentandra
Trianthema_portulacastrum_SRA
Kewa_bowkeriana
Kewa_caespitosa
Limeum_aethiopicum
Grayia_brandegeei
Grayia_spinosa
Extriplex_californica
Stutzia_covillei
Proatriplex_pleiantha
Atriplex_prostrata
Atriplex_hortensis
Atriplex_rosea
Atriplex_sp
Chenopodium_ficifolium
Chenopodium_suecicum
Chenopodium_giganteum
Chenopodium_quinoa
Chenopodium_pallidicaule
Chenopodiastrum_murale
Oxybasis_rubra
Spinacia_turkestanica
Spinacia_tetrandra
Spinacia_oleracea
Krascheninnikovia_lanata
Suckleya_suckleyana
Dysphania_schraderiana
Agriophyllum_squarrosum
Corispermum_hyssopifolium
Beta_maritima
Beta_vulgaris
Beta_patula
Tecticornia_pergranulata
Salicornia_brachiata
Salicornia_europaea
Salicornia_pacifica
Arthrocnemum_macrostachyum
Kalidium_cuspidatum
Halostachys_caspica
Salsola_sp_SAMEAS
Suaeda_maritima
Suaeda_salsa
Suaeda_linearis
Suaeda_fruticosa
Suaeda_aralocaspica
Bienertia_sinuspersici
Haloxylon_ammodendron
Hammada_scoparia
Halogeton_glomeratus
Anabasis_articulata
Salsola_collina
Caroxylon_vermiculatum
Eokochia_saxicola
Bassia_scoparia
Polycnemum_majus
Nitrophila_occidentalis
Alternanthera_philoxeroides
Alternanthera_brasiliana
Alternanthera_sessilis
Alternanthera_caracasana
Alternanthera_ficoidea
Tidestromia_lanuginosa
Blutaparon_vermiculare_SYN
Gossypianthus_lanuginosus
Guilleminea_densa_var_aggregata
Froelichia_floridana
Achyranthes_bidentata
Nelsia_quadrangula
Aerva_lanata
Ptilotus_exaltatus_hyb
Aerva_javanica_SYN
Iresine_rhizomatosa
Iresine_arbuscula
Amaranthus_retroflexus
Amaranthus_hypochondriacus
Amaranthus_palmeri
Amaranthus_tuberculatus
Amaranthus_tricolor
Amaranthus_cruentus
Phaulothamnus_spinescens_SYN
Achatocarpus_gracilis
Cerastium_alpinum
Cerastium_arvense
Cerastium_fontanum_subsp_vulgare
Stellaria_aquatica
Pseudostellaria_heterophylla
Lepyrodiclis_stellarioides
Arenaria_serpyllifolia
Schiedea_globosa
Schiedea_membranacea
Honckenya_peploides
Scleranthus_polycarpos
Colobanthus_quitensis
Dianthus_caryophyllus
Dianthus_superbus
Velezia_rigida
Gypsophila_repens
Saponaria_officinalis
Silene_noctiflora
Silene_conica
Silene_latifolia
Silene_vulgaris
Silene_acaulis_ssp_acaulescens
Silene_paradoxa
Agrostemma_githago
Eremogone_procera
Eremogone_hookeri
Spergularia_media
Spergularia_marina
Spergula_arvensis
Cardionema_ramosissimum
Illecebrum_verticillatum
Drymaria_subumbellata
Drymaria_cordata
Polycarpon_tetraphyllum
Pollichia_campestris
Polycarpaea_repens
Paronychia_fastigiata
Paronychia_drummondii
Paronychia_jamesii
Gymnocarpos_przewalskii
Herniaria_glabra
Herniaria_latifolia
Telephium_imperatii
Corrigiola_litoralis
Stegnosperma_halimifolium
Macarthuria_australis
Microtea_debilis
Physena_madagascariensis
Simmondsia_chinensis
Rheum_nobile
Rheum_rhomboideum
Rheum_rhabarbarum
Oxyria_digyna
Rumex_hastatulus
Rumex_acetosa
Rumex_palustris
Emex_spinosa
Fallopia_sachalinensis
Reynoutria_japonica
Fallopia_convolvulus
Muehlenbeckia_platyclada
Polygonum_dentoceras
Polygonum_aviculare
Fagopyrum_tataricum
Fagopyrum_esculentum
Persicaria_minor
Persicaria_tinctoria
Persicaria_virginiana
Bistorta_bistortoides
Eriogonum_inflatum
Eriogonum_arcuatum
Eriogonum_longifolium
Eriogonum_rotundifolium
Eriogonum_deflexum
Eriogonum_callistum
Chorizanthe_angustifolia
Oxytheca_perfoliata
Sidotheca_caryophylloides
Stenogonum_salsuginosum
Dedeckera_eurekensis
Pterostegia_drymarioides
Ruprechtia_salicifolia
Ruprechtia_coriacea
Triplaris_weigeltiana
Podopterus_cordifolius
Podopterus_mexicanus
Coccoloba_uvifera
Antigonon_leptopus
Limonium_spectabile
Limonium_californicum
Acantholimon_lycopodioides
Aegialitis_annulata
Plumbago_auriculata
Reaumuria_trigyna
Reaumuria_songarica_SYN
Tamarix_hispida
Tamarix_chinensis
Frankenia_salina
Frankenia_laevis
Drosera_capensis
Drosera_spatulata
Drosera_burmannii
Drosera_binata
Dionaea_muscipula
Aldrovanda_vesiculosa
Nepenthes_ventricosa
Nepenthes_alata
Nepenthes_ampullaria
Ancistrocladus_robertsonianum
Drosophyllum_lusitanicum

Figure S2: Maximum likelihood reconciliation of DODAα sequences from GeneRax. Branches that were adjusted in the final tree are highlighted in red. Scale bar gives 0.1 expected substitutions per site.

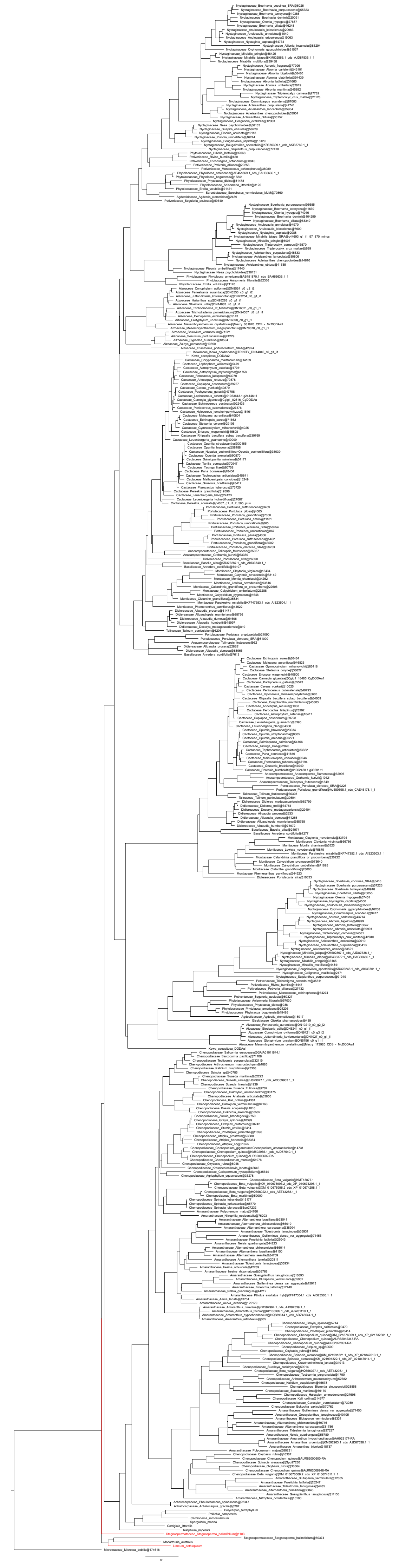

Figure S3: Modified reconciled topology. DODAα1 reconciliation topology after adjusting the position of sequences implying unparsimonious losses to match the species tree topology. Branch lengths in expected substitutions per site, scale bar gives 0.1 substitutions per site.

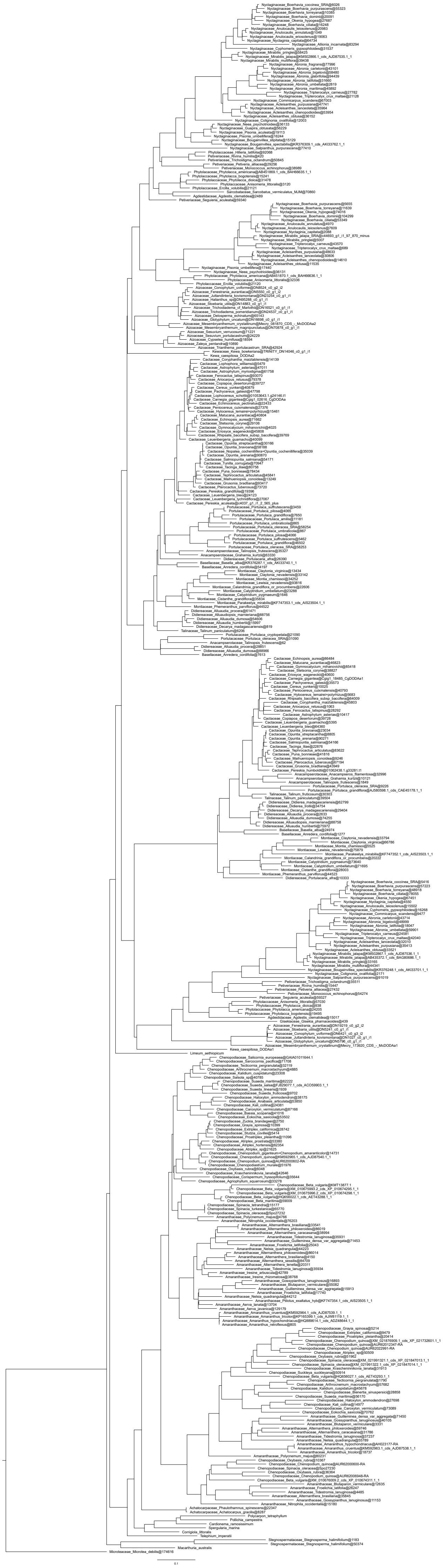

Figure S4: Final pruned reconciled topology. DODAα1 modified reconciliation topology after removing fragmentary sequences and inferring maximum likelihood branch lengths under JTT+G. This topology and branch lengths were used for ancestral sequence reconstruction and convergence analyses. Branch lengths in expected substitutions per site. Scale bar gives 0.2 expected substitutions per site.

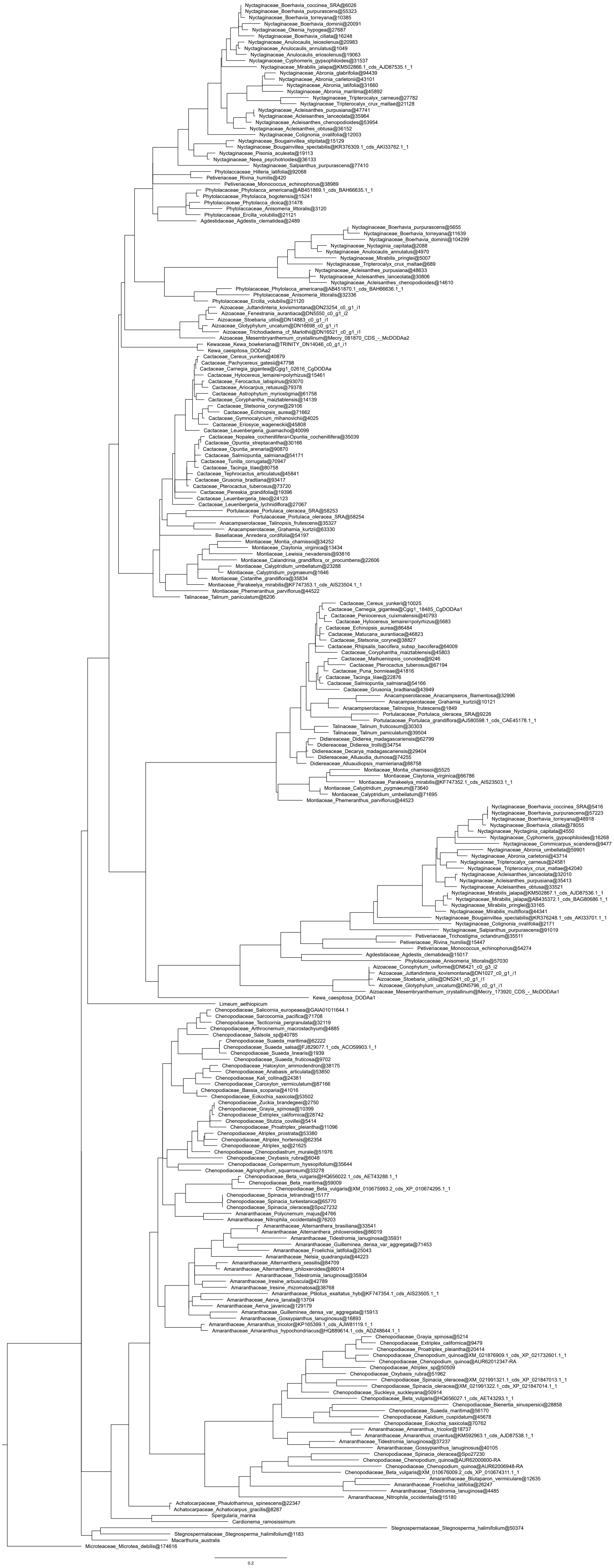

Nyctaginaceae_Boerhavia_coccinea_SRA@6026
Nyctaginaceae_Boerhavia_purpurascens@55323
Nyctaginaceae_Boerhavia_torreyana@10385
Nyctaginaceae_Boerhavia_dominii@20091
Nyctaginaceae_Okenia_hypogea@27687
Nyctaginaceae_Boerhavia_ciliata@16248
Nyctaginaceae_Anulocaulis_leiosolenus@20983
Nyctaginaceae_Anulocaulis_annulatus@1049
Nyctaginaceae_Anulocaulis_eriosolenus@19063
Nyctaginaceae_Cyphomeris_gypsophiloides@31537
Nyctaginaceae_Mirabilis_jalapa@KM502866.1_cds_AJD87535.1_1
Nyctaginaceae_Abronia_glabrifolia@94439
Nyctaginaceae_Abronia_carletonii@43101
Nyctaginaceae_Abronia_latifolia@31660
Nyctaginaceae_Abronia_maritima@45892
Nyctaginaceae_Tripterocalyx_carneus@27782
Nyctaginaceae_Tripterocalyx_crux_maltae@21128
Nyctaginaceae_Acleisanthes_purpusiana@47741
Nyctaginaceae_Acleisanthes_lanceolata@35964
Nyctaginaceae_Acleisanthes_chenopodioides@53954
Nyctaginaceae_Acleisanthes_obtusa@36152
Nyctaginaceae_Colignonia_ovalifolia@12003
Nyctaginaceae_Bougainvillea_stipitata@15129
Nyctaginaceae_Bougainvillea_spectabilis@KR376309.1_cds_AKI33762.1_1
Nyctaginaceae_Pisonia_aculeata@19113
Nyctaginaceae_Neea_psychotrioides@36133
Nyctaginaceae_Salpianthus_purpurascens@77410
Phytolaccaceae_Hilleria_latifolia@92068
Petiveriaceae_Rivina_humilis@420
Petiveriaceae_Monococcus_echinophorus@38989
Phytolaccaceae_Phytolacca_americana@AB451869.1_cds_BAH66635.1_1
Phytolaccaceae_Phytolacca_bogotensis@15241
Phytolaccaceae_Phytolacca_dioica@31478
Phytolaccaceae_Anisomeria_littoralis@3120
Phytolaccaceae_Ercilla_volubilis@21121
Agdestidaceae_Agdestis_clematidea@2489
Nyctaginaceae_Boerhavia_purpurascens@5655
Nyctaginaceae_Boerhavia_torreyana@11639
Nyctaginaceae_Boerhavia_dominii@104299
Nyctaginaceae_Nyctaginia_capitata@2088
Nyctaginaceae_Anulocaulis_annulatus@4970
Nyctaginaceae_Mirabilis_pringlei@5007
Nyctaginaceae_Tripterocalyx_crux_maltae@689
Nyctaginaceae_Acleisanthes_purpusiana@48633
Nyctaginaceae_Acleisanthes_lanceolata@30806
Nyctaginaceae_Acleisanthes_chenopodioides@14610
Phytolacaceae_Phytolacca_americana@AB451870.1_cds_BAH66636.1_1
Phytolaccaceae_Anisomeria_littoralis@32336
Phytolaccaceae_Ercilla_volubilis@21120
Aizoaceae_Juttandinteria_kovismontana@DN23254_c0_g1_i1
Aizoaceae_Fenestrania_aurantiaca@DN5550_c0_g1_i2
Aizoaceae_Stoebaria_utilis@DN14883_c0_g1_i1
Aizoaceae_Glotyphylum_uncatum@DN16698_c0_g1_i1
Aizoaceae_Trichodiadema_cf_Marlothii@DN16521_c0_g1_i1
Aizoaceae_Mesembryanthemum_crystallinum@Mecry_081870_CDS_-_McDODAa2
Kewaceae_Kewa_bowkeriana@TRINITY_DN14046_c0_g1_i1
Kewa_caespitosa_DODAa2
Cactaceae_Cereus_yunkeri@40879
Cactaceae_Pachycereus_gatesii@47798
Cactaceae_Carnegia_gigantea@Cgig1_02616_CgDODAa
Cactaceae_Hylocereus_lemairei=polyrhizus@15461
Cactaceae_Ferocactus_latispinus@93070
Cactaceae_Ariocarpus_retusus@79378
Cactaceae_Astrophytum_myriostigma@61758
Cactaceae_Coryphantha_maiztablensis@14139
Cactaceae_Stetsonia_coryne@29106
Cactaceae_Echinopsis_aurea@71662
Cactaceae_Gymnocalycium_mihanovichii@4025
Cactaceae_Eriosyce_wageneckii@45808
Cactaceae_Leuenbergeria_guamacho@40099
Cactaceae_Nopalea_cochenillifera=Opuntia_cochenillifera@35039
Cactaceae_Opuntia_streptacantha@30166
Cactaceae_Opuntia_arenaria@90870
Cactaceae_Salmiopuntia_salmiana@54171
Cactaceae_Tunilla_corrugata@70947
Cactaceae_Tacinga_lilae@80758
Cactaceae_Tephrocactus_articulatus@45841
Cactaceae_Grusonia_bradtiana@93417
Cactaceae_Pterocactus_tuberosus@73720
Cactaceae_Pereskia_grandifolia@19396
Cactaceae_Leuenbergeria_bleo@24123
Cactaceae_Leuenbergeria_lychnidiflora@27067
Portulacaceae_Portulaca_oleracea_SRA@58253
Portulacaceae_Portulaca_oleracea_SRA@58254
Anacampserotaceae_Talinopsis_frutescens@35327
Anacampserotaceae_Grahamia_kurtzii@63330
Basellaceae_Anredera_cordifolia@54197
Montiaceae_Montia_chamissoi@34252
Montiaceae_Claytonia_virginica@13434
Montiaceae_Lewisia_nevadensis@93816
Montiaceae_Calandrinia_grandiflora_or_procumbens@22606
Montiaceae_Calyptridium_umbellatum@23288
Montiaceae_Calyptridium_pygmaeum@1646
Montiaceae_Cistanthe_grandiflora@35834
Montiaceae_Parakeelya_mirabilis@KF747353.1_cds_AIS23504.1_1
Montiaceae_Phemeranthus_parviflorus@44522
Talinaceae_Talinum_paniculatum@6206
Cactaceae_Cereus_yunkeri@10025
Cactaceae_Carnegia_gigantea@Cgig1_18485_CgDODAa1
Cactaceae_Peniocereus_cuixmalensis@40793
Cactaceae_Hylocereus_lemairei=polyrhizus@5683
Cactaceae_Echinopsis_aurea@86484
Cactaceae_Matucana_aurantiaca@46823
Cactaceae_Stetsonia_coryne@38827
Cactaceae_Rhipsalis_baccifera_subsp_baccifera@64009
Cactaceae_Coryphantha_maiztablensis@45803
Cactaceae_Maihueniopsis_conoidea@9246
Cactaceae_Pterocactus_tuberosus@67194
Cactaceae_Puna_bonnieae@41816
Cactaceae_Tacinga_lilae@22876
Cactaceae_Salmiopuntia_salmiana@54166
Cactaceae_Grusonia_bradtiana@43949
Anacampserotaceae_Anacampseros_filamentosa@32996
Anacampserotaceae_Grahamia_kurtzii@10121
Anacampserotaceae_Talinopsis_frutescens@1849
Portulacaceae_Portulaca_oleracea_SRA@9226
Portulacaceae_Portulaca_grandiflora@AJ580598.1_cds_CAE45178.1_1
Talinaceae_Talinum_fruticosum@30303
Talinaceae_Talinum_paniculatum@39504
Didiereaceae_Didierea_madagascariensis@62799
Didiereaceae_Didierea_trollii@34754
Didiereaceae_Decarya_madagascariensis@29404
Didiereaceae_Alluaudia_dumosa@74255
Didiereaceae_Alluaudiopsis_marnieriana@88758
Montiaceae_Montia_chamissoi@5525
Montiaceae_Claytonia_virginica@66786
Montiaceae_Parakeelya_mirabilis@KF747352.1_cds_AIS23503.1_1
Montiaceae_Calyptridium_pygmaeum@73640
Montiaceae_Calyptridium_umbellatum@71695
Montiaceae_Phemeranthus_parviflorus@44523
Nyctaginaceae_Boerhavia_coccinea_SRA@5416
Nyctaginaceae_Boerhavia_purpurascens@57223
Nyctaginaceae_Boerhavia_torreyana@48918
Nyctaginaceae_Boerhavia_ciliata@78055
Nyctaginaceae_Nyctaginia_capitata@4550
Nyctaginaceae_Cyphomeris_gypsophiloides@16268
Nyctaginaceae_Commicarpus_scandens@9477
Nyctaginaceae_Abronia_umbellata@59901
Nyctaginaceae_Abronia_carletonii@43714
Nyctaginaceae_Tripterocalyx_carneus@24581
Nyctaginaceae_Tripterocalyx_crux_maltae@42040
Nyctaginaceae_Acleisanthes_lanceolata@32010
Nyctaginaceae_Acleisanthes_purpusiana@35413
Nyctaginaceae_Acleisanthes_obtusa@33521
Nyctaginaceae_Mirabilis_jalapa@KM502867.1_cds_AJD87536.1_1
Nyctaginaceae_Mirabilis_jalapa@AB435372.1_cds_BAG80686.1_1
Nyctaginaceae_Mirabilis_pringlei@33165
Nyctaginaceae_Mirabilis_multiflora@44341
Nyctaginaceae_Bougainvillea_spectabilis@KR376248.1_cds_AKI33701.1_1
Nyctaginaceae_Colignonia_ovalifolia@2171
Nyctaginaceae_Salpianthus_purpurascens@91019
Petiveriaceae_Trichostigma_octandrum@35511
Petiveriaceae_Rivina_humilis@15447
Petiveriaceae_Monococcus_echinophorus@54274
Agdestidaceae_Agdestis_clematidea@15017
Phytolaccaceae_Anisomeria_littoralis@57030
Aizoaceae_Conophytum_uviforme@DN6421_c0_g3_i2
Aizoaceae_Juttandinteria_kovismontana@DN1027_c0_g1_i1
Aizoaceae_Stoebaria_utilis@DN5241_c0_g1_i1
Aizoaceae_Glotyphylum_uncatum@DN5796_c0_g1_i1
Aizoaceae_Mesembryanthemum_crystallinum@Mecry_173920_CDS_-_McDODAa1
Kewa_caespitosa_DODAa1
Limeum_aethiopicum
Chenopodiaceae_Salicornia_europeaea@GAIA01011644.1
Chenopodiaceae_Sarcocornia_pacifica@71708
Chenopodiaceae_Tecticornia_pergranulata@32119
Chenopodiaceae_Arthrocnemum_macrostachyum@4885
Chenopodiaceae_Salsola_sp@40785
Chenopodiaceae_Suaeda_maritima@62222
Chenopodiaceae_Suaeda_salsa@FJ829077.1_cds_ACO59903.1_1
Chenopodiaceae_Suaeda_linearis@1939
Chenopodiaceae_Suaeda_fruticosa@9702
Chenopodiaceae_Haloxylon_ammodendron@38175
Chenopodiaceae_Anabasis_articulata@53850
Chenopodiaceae_Kali_collina@24381
Chenopodiaceae_Caroxylon_vermiculatum@87166
Chenopodiaceae_Bassia_scoparia@41016
Chenopodiaceae_Eokochia_saxicola@53502
Chenopodiaceae_Zuckia_brandegeei@2750
Chenopodiaceae_Grayia_spinosa@10399
Chenopodiaceae_Extriplex_californica@28742
Chenopodiaceae_Stutzia_covillei@5414
Chenopodiaceae_Proatriplex_pleiantha@11096
Chenopodiaceae_Atriplex_prostrata@53380
Chenopodiaceae_Atriplex_hortensis@62354
Chenopodiaceae_Atriplex_sp@21625
Chenopodiaceae_Chenopodiastrum_murale@51976
Chenopodiaceae_Oxybasis_rubra@6048
Chenopodiaceae_Corispermum_hyssopifolium@35644
Chenopodiaceae_Agriophyllum_squarrosum@33278
Chenopodiaceae_Beta_vulgaris@HQ656022.1_cds_AET43288.1_1
Chenopodiaceae_Beta_maritima@59009
Chenopodiaceae_Beta_vulgaris@XM_010675993.2_cds_XP_010674295.1_1
Chenopodiaceae_Spinacia_tetrandra@15177
Chenopodiaceae_Spinacia_turkestanica@65770
Chenopodiaceae_Spinacia_oleracea@Spo27232
Amaranthaceae_Polycnemum_majus@4766
Amaranthaceae_Nitrophila_occidentalis@76203
Amaranthaceae_Alternanthera_brasiliana@33541
Amaranthaceae_Alternanthera_philoxeroides@86019
Amaranthaceae_Tidestromia_lanuginosa@35931
Amaranthaceae_Guilleminea_densa_var_aggregata@71453
Amaranthaceae_Froelichia_latifolia@25043
Amaranthaceae_Nelsia_quadrangula@44223
Amaranthaceae_Alternanthera_sessilis@84709
Amaranthaceae_Alternanthera_philoxeroides@86014
Amaranthaceae_Tidestromia_lanuginosa@35934
Amaranthaceae_Iresine_arbuscula@42789
Amaranthaceae_Iresine_rhizomatosa@38768
Amaranthaceae_Ptilotus_exaltatus_hyb@KF747354.1_cds_AIS23505.1_1
Amaranthaceae_Aerva_lanata@13704
Amaranthaceae_Aerva_javanica@129179
Amaranthaceae_Guilleminea_densa_var_aggregata@15913
Amaranthaceae_Gossypianthus_lanuginosus@16893
Amaranthaceae_Amaranthus_tricolor@KP165399.1_cds_AJW81119.1_1
Amaranthaceae_Amaranthus_hypochondriacus@HQ889614.1_cds_ADZ48644.1_1
Chenopodiaceae_Grayia_spinosa@5214
Chenopodiaceae_Extriplex_californica@9479
Chenopodiaceae_Proatriplex_pleiantha@20414
Chenopodiaceae_Chenopodium_quinoa@XM_021876909.1_cds_XP_021732601.1_1
Chenopodiaceae_Chenopodium_quinoa@AUR62012347-RA
Chenopodiaceae_Atriplex_sp@50509
Chenopodiaceae_Oxybasis_rubra@51962
Chenopodiaceae_Spinacia_oleracea@XM_021991321.1_cds_XP_021847013.1_1
Chenopodiaceae_Spinacia_oleracea@XM_021991322.1_cds_XP_021847014.1_1
Chenopodiaceae_Suckleya_suckleyana@50914
Chenopodiaceae_Beta_vulgaris@HQ656027.1_cds_AET43293.1_1
Chenopodiaceae_Bienertia_sinuspersici@28858
Chenopodiaceae_Suaeda_maritima@56170
Chenopodiaceae_Kalidium_cuspidatum@45678
Chenopodiaceae_Eokochia_saxicola@70762
Amaranthaceae_Amaranthus_tricolor@18737
Amaranthaceae_Amaranthus_cruentus@KM592963.1_cds_AJD87538.1_1
Amaranthaceae_Tidestromia_lanuginosa@37237
Amaranthaceae_Gossypianthus_lanuginosus@40105
Chenopodiaceae_Spinacia_oleracea@Spo27230
Chenopodiaceae_Chenopodium_quinoa@AUR62000600-RA
Chenopodiaceae_Chenopodium_quinoa@AUR62006948-RA
Chenopodiaceae_Beta_vulgaris@XM_010676009.2_cds_XP_010674311.1_1
Amaranthaceae_Blutaparon_vermiculare@12635
Amaranthaceae_Froelichia_latifolia@26247
Amaranthaceae_Tidestromia_lanuginosa@4485
Amaranthaceae_Nitrophila_occidentalis@15180
Achatocarpaceae_Phaulothamnus_spinescens@22347
Achatocarpaceae_Achatocarpus_gracilis@8287
Spergularia_marina
Cardionema_ramosissimum
Stegnospermataceae_Stegnosperma_halimifolium@50374
Stegnospermataceae_Stegnosperma_halimifolium@1183
Macarthuria_australis
Microteaceae_Microtea_debilis@174616
0.2

Figure S5: A paralogue of Spinach DODAα1 shows low activity. a) subtree of Amaranthaceae DODAα1 extracted from the reconciled topology showing the positions of two paralogues from Spinacia oleracea and Beta vulgaris DODAα1. b) fluorescence of both spinach paralogues expressed in yeast compared to BvDODAα1. c) multiple sequence alignment of the three sequences (accessions as in the tips of the tree in a) showing differences between the two paralogues. Substitutions overlapping with any unique, divergent or convergent substitution inferred from our ancestral sequence reconstructions are labelled.

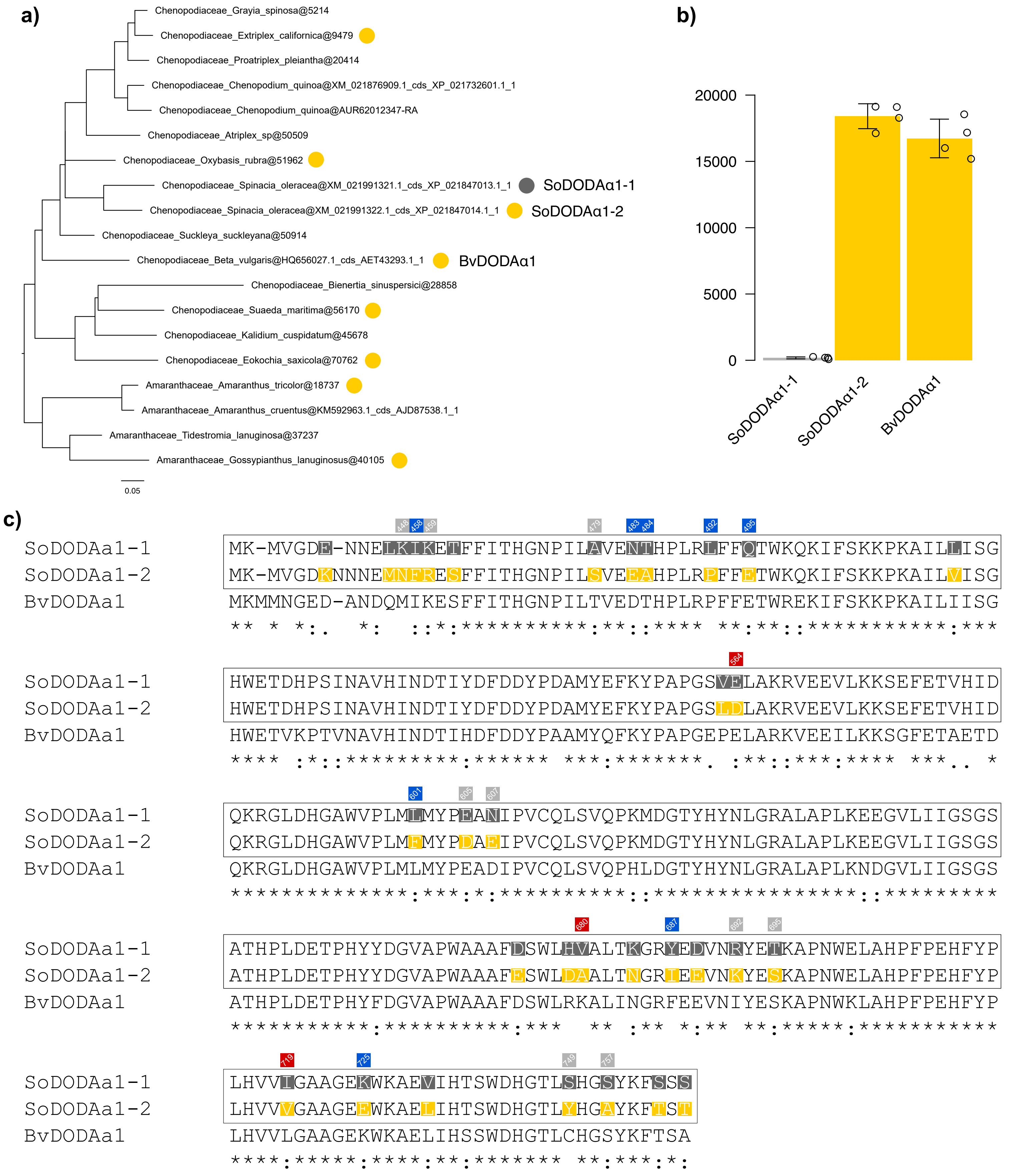
a)
Chenopodiaceae_Grayia_spinosa@5214
Chenopodiaceae_Extriplex_californica@9479
Chenopodiaceae_Proatriplex_pleiantha@20414
Chenopodiaceae_Chenopodium_quinoa@XM_021876909.1_cds_XP_021732601.1_1
Chenopodiaceae_Chenopodium_quinoa@AUR62012347-RA
Chenopodiaceae_Atriplex_sp@50509
Chenopodiaceae_Oxybasis_rubra@51962
Chenopodiaceae_Spinacia_oleracea@XM_021991321.1_cds_XP_021847013.1_1
SoDODAα1-1
Chenopodiaceae_Spinacia_oleracea@XM_021991322.1_cds_XP_021847014.1_1
SoDODAα1-2
Chenopodiaceae_Suckleya_suckleyana@50914
Chenopodiaceae_Beta_vulgaris@HQ656027.1_cds_AET43293.1_1
BvDODAα1
Chenopodiaceae_Bienertia_sinuspersici@28858
Chenopodiaceae_Suaeda_maritima@56170
Chenopodiaceae_Kalidium_cuspidatum@45678
Chenopodiaceae_Eokochia_saxicola@70762
Amaranthaceae_Amaranthus_tricolor@18737
Amaranthaceae_Amaranthus_cruentus@KM592963.1_cds_AJD87538.1_1
Amaranthaceae_Tidestromia_lanuginosa@37237
Amaranthaceae_Gossypianthus_lanuginosus@40105
0.05
b)
20000
15000
10000
5000
0
SoDODAα1-1
SoDODAα1-2
BvDODAα1
c)
SoDODAa1-1 MK-MVGDE-NNELKIKETFFITHGNPILAVENTHPLRLFFQTWKQKIFSKKPKAILLISG
SoDODAa1-2 MK-MVGDKNNNEMNFRESFFITHGNPILSVEEAHPLRPFFETWKQKIFSKKPKAILVISG
BvDODAa1 MKMMNGED-ANDQMIKESFFITHGNPILTVEDTHPLRPFFETWREKIFSKKPKAILIISG
SoDODAa1-1 HWETDHPSINAVHINDTIYDFDDYPDAMYEFKYPAPGSVELAKRVEEVLKKSEFETVHID
SoDODAa1-2 HWETDHPSINAVHINDTIYDFDDYPDAMYEFKYPAPGSLDLAKRVEEVLKKSEFETVHID
BvDODAa1 HWETVKPTVNAVHINDTIHDFDDYPAAMYQFKYPAPGEPELARKVEEILKKSGFETAETD
SoDODAa1-1 QKRGLDHGAWVPLMLMYPEANIPVCQLSVQPKMDGTYHYNLGRALAPLKEEGVLIIGSGS
SoDODAa1-2 QKRGLDHGAWVPLMEMYPDAEIPVCQLSVQPKMDGTYHYNLGRALAPLKEEGVLIIGSGS
BvDODAa1 QKRGLDHGAWVPLMLMYPEADIPVCQLSVQPHLDGTYHYNLGRALAPLKNDGVLIIGSGS
SoDODAa1-1 ATHPLDETPHYYDGVAPWAAAFDSWLHVALTKGRYEDVNRYETKAPNWELAHPFPEHFYP
SoDODAa1-2 ATHPLDETPHYYDGVAPWAAAFESWLDAALTNGRIEEVNKYESKAPNWELAHPFPEHFYP
BvDODAa1 ATHPLDETPHYFDGVAPWAAAFDSWLRKALINGRFEEVNIYESKAPNWKLAHPFPEHFYP
SoDODAa1-1 LHVVIGAAGEKWKAEVIHTSWDHGTLSHGSYKFSSS
SoDODAa1-2 LHVVVGAAGEEWKAELIHTSWDHGTLYHGAYKFTST
BvDODAa1 LHVVLGAAGEKWKAELIHSSWDHGTLCHGSYKFTSA

Figure S6: Posterior probabilities of reconstructed sequences. Each histogram represents a single reconstructed node, showing the frequency of posterior probabilities for the maximum posterior probability state in each site.

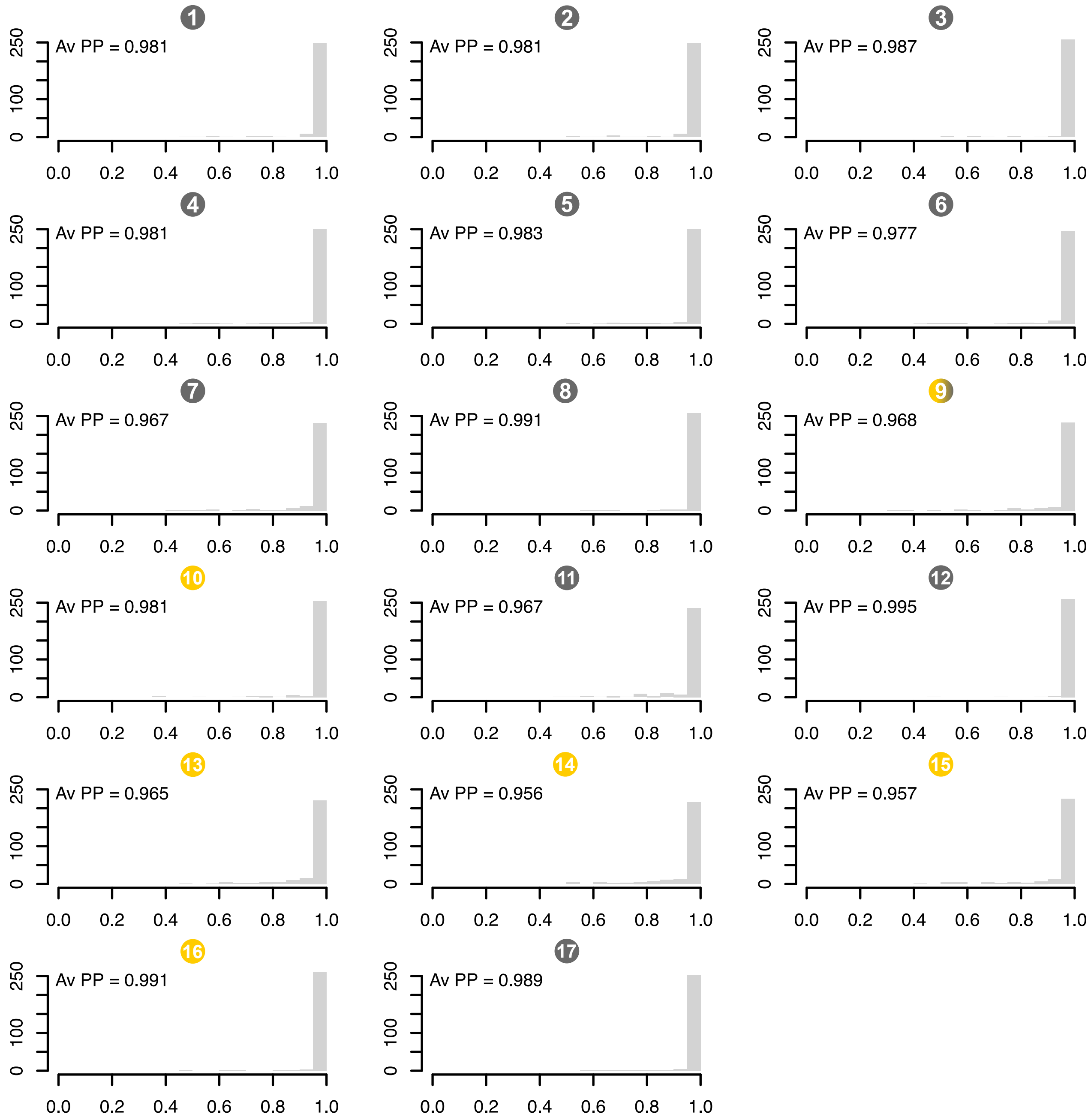

Figure S7: Expected convergence and divergence based on parametric bootstrapping with simulation. Branch or lineage pairs that are not sister or directly ancestral or descended are compared. Each cell gives the observed value, expected value (calculated by dividing the count of the observed event by the number of simulation replicates, 1000), ratio, and P-value (computed by counting the proportion of simulations showing the same or a more extreme result). The lower half gives convergence, upper half gives divergence. Significant convergent results are highlighted in red and significant divergent results in blue. Results from comparing states at the end of **a)** lineages **b)** branches.

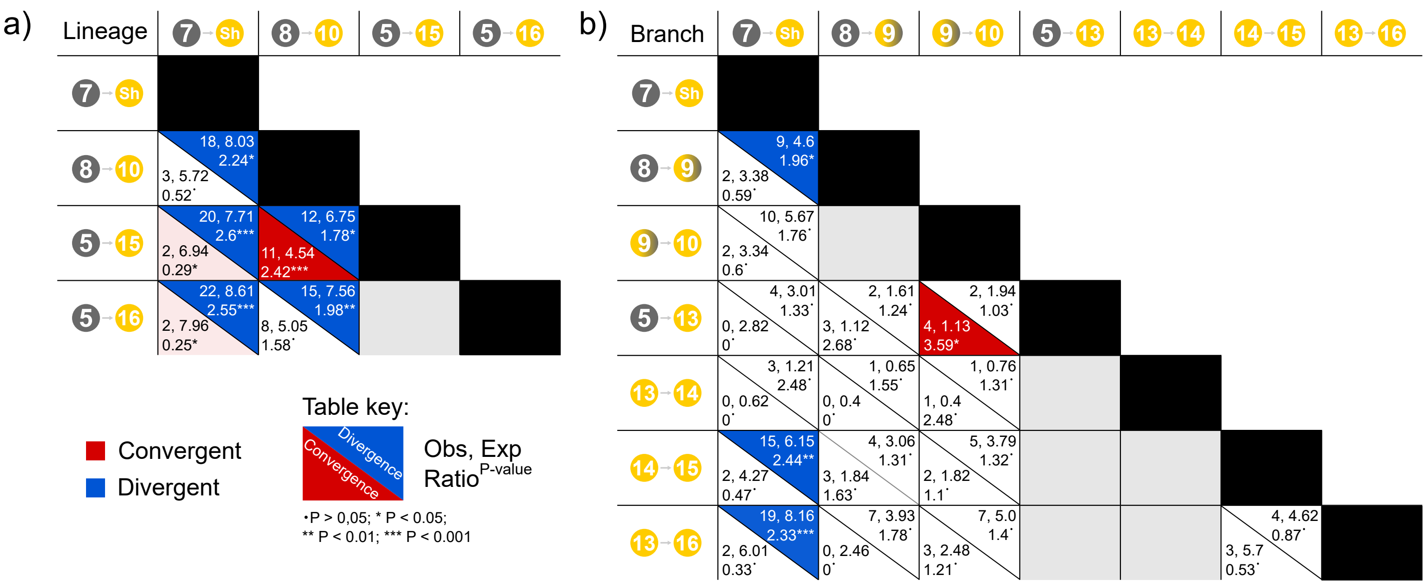

Figure S8: Structural context of inferred substitutions per branch. Branch substitutions (comparing ancestor and descendant of each branch) mapped against proximity to the binding pocket centroid and extant divergence and conservation in the alignment. **a)** predicted protein structure surfaces for each reconstructed descendant sequence (extant observed sequence for *Sh*DODAα1) from AlphaFold2, with residues coloured according to whether they contain an inferred convergent, divergent, or unique substitution along their subtending branch. **b)** combined divergence of site-specific amino acid frequencies between and conservation within DODAα1 and DODAα2 clades descended from each branch at substituting sites, displayed on the structure of the descendant node in each comparison.

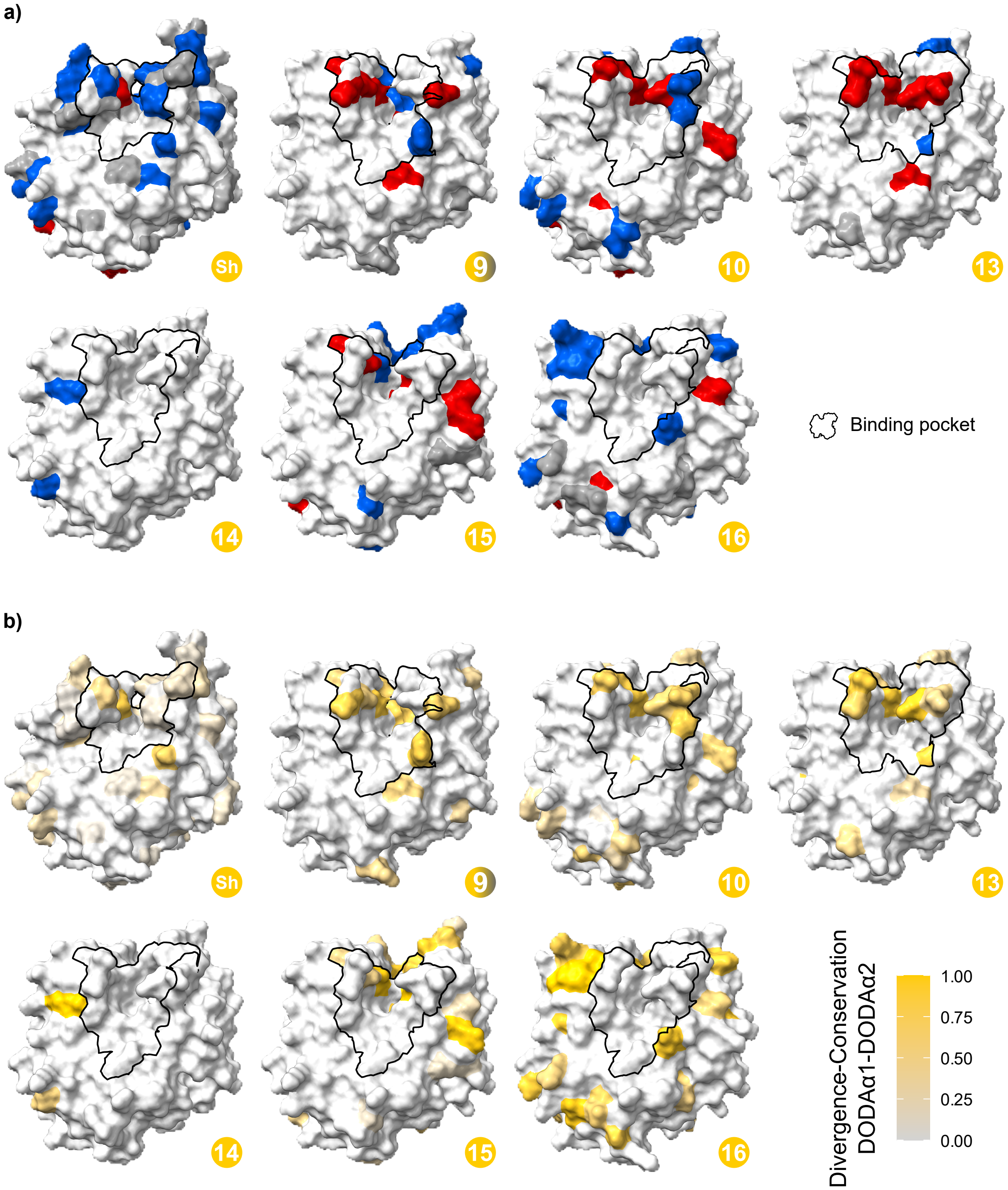
a)
Sh
9
10
13
14
15
16
Binding pocket
b)
Sh
9
10
13
14
15
16
Divergence-Conservation
DODAα1-DODAα2
1.00
0.75
0.50
0.25
0.00

Figure S9: Comparison of states in ShDODAα1, ShDODAα2, and inferred ancestors. Multiple sequence alignment of extant sequences and reconstructed ancestors extracted from the larger alignment used for ancestral sequence reconstruction. Derived DODAα1-like states are highlighted in gold, DODAα1-DODAα2 ancestor-like states in grey, and alternative states in black. Convergent, divergent or unique substitutions inferred from our ancestral sequence reconstructions are labelled.

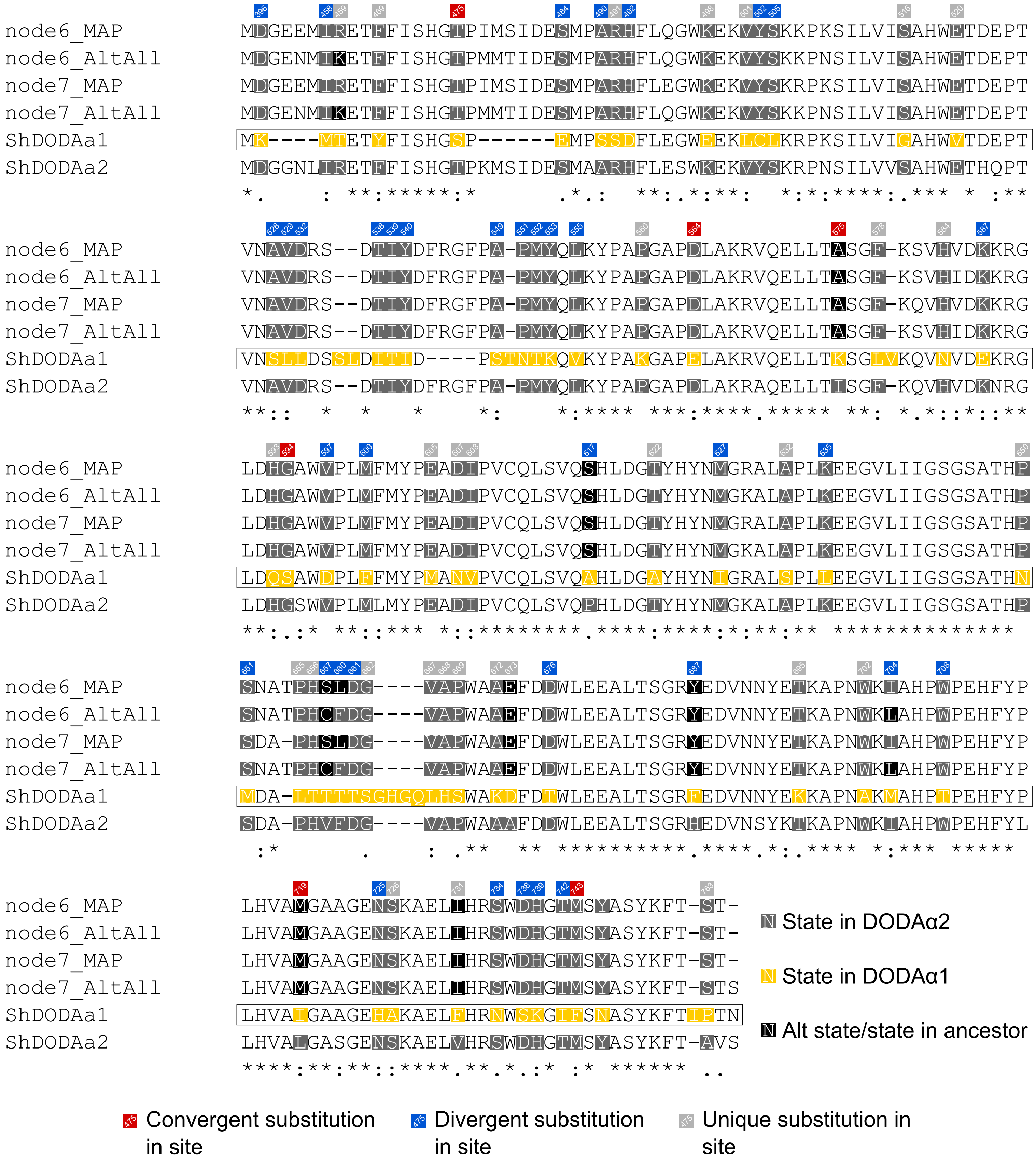

node6_MAP MDGEEMIRETFFISHGTPIMSIDESMPARHFLQGWKEKVYSKKPKSILVISAHWETDEPT
node6_AltAll MDGENMIKETFFISHGTPMMTIDESMPARHFLQGWKEKVYSKKPNSILVISAHWETDEPT
node7_MAP MDGEEMIRETFFISHGTPIMSIDESMPARHFLEGWKEKVYSKRPKSILVISAHWETDEPT
node7_AltAll MDGENMIKETFFISHGTPMMTIDESMPARHFLQGWKEKVYSKKPNSILVISAHWETDEPT
ShDODAa1 MK----MTETYFISHGSP------EMPSSDFLEGWEEKLCLKRPKSILVIGAHWVTDEPT
ShDODAa2 MDGGNLIRETFFISHGTPKMSIDESMAARHFLESWKEKVYSKRPNSILVVSAHWETHQPT
*.   : **:*****:*   .*.: **:.*:**: *:*:****:.*** * :**
node6_MAP VNAVDRS--DTIYDFRGFPA-PMYQLKYPAPGAPDLAKRVQELLTASGE-KSVHVDKKRG
node6_AltAll VNAVDRS--DTIYDFRGFPA-PMYQLKYPAPGAPDLAKRVQELLTASGE-KSVHIDKKRG
node7_MAP VNAVDRS--DTIYDFRGFPA-PMYQLKYPAPGAPDLAKRVQELLTASGE-KQVHVDKKRG
node7_AltAll VNAVDRS--DTIYDFRGFPA-PMYQLKYPAPGAPDLAKRVQELLTASGE-KSVHIDKKRG
ShDODAa1 VNSLLDSSLDITID----PSTNTKQVKYPAKGAPELAKRVQELLTKSGLVKQVNVDEKRG
ShDODAa2 VNAVDRS--DTIYDFRGFPA-PMYQLKYPAPGAPDLAKRAQELLTISGE-KQVHVDKNRG
:: * *   *   *:   *:** ***:****.***** **: *.*::*::**
node6_MAP LDHGAWVPLMFMYPEADIPVCQLSVQSHLDGTYHYNMGRALAPLKEEGVLIIGSGSATHP
node6_AltAll LDHGAWVPLMFMYPEADIPVCQLSVQSHLDGTYHYNMGKALAPLKEEGVLIIGSGSATHP
node7_MAP LDHGAWVPLMFMYPEADIPVCQLSVQSHLDGTYHYNMGRALAPLKEEGVLIIGSGSATHP
node7_AltAll LDHGAWVPLMFMYPEADIPVCQLSVQSHLDGTYHYNMGKALAPLKEEGVLIIGSGSATHP
ShDODAa1 LDQSAWDPLFFMYPMANVPVCQLSVQAHLDGAYHYNIGRALSPLLEEGVLIIGSGSATHN
ShDODAa2 LDHGSWVPLMLMYPEADIPVCQLSVQPHLDGTYHYNMGKALAPLKEEGVLIIGSGSATHP
:.:* **::* *::********.****:****:*:**:** **************
node6_MAP SNATPHSLDG----VAPWAAEFDDWLEEALTSGRYEDVNNYETKAPNWKIAHPWPEHFYP
node6_AltAll SNATPHCFDG----VAPWAAEFDDWLEEALTSGRYEDVNNYETKAPNWKLAHPWPEHFYP
node7_MAP SDA-PHSLDG----VAPWAAEFDDWLEEALTSGRYEDVNNYETKAPNWKIAHPWPEHFYP
node7_AltAll SNATPHCFDG----VAPWAAEFDDWLEEALTSGRYEDVNNYETKAPNWKLAHPWPEHFYP
ShDODAa1 MDA-LTTTTSGHGQLHSWAKDFDTWLEEALTSGRFEDVNNYEKKAPNAKMAHPTPEHFYP
ShDODAa2 SDA-PHVFDG----VAPWAAAFDDWLEEALTSGRHEDVNSYKTKAPNWKIAHPWPEHFYL
:*   .   : .**   ** **********.****.*:.**** *:*** *****
node6_MAP LHVAMGAAGENSKAELIHRSWDHGTMSYASYKFT-ST-
node6_AltAll LHVAMGAAGENSKAELIHRSWDHGTMSYASYKFT-ST-
node7_MAP LHVAMGAAGENSKAELIHRSWDHGTMSYASYKFT-ST-
node7_AltAll LHVAMGAAGENSKAELIHRSWDHGTMSYASYKFT-STS
ShDODAa1 LHVAIGAAGEHAKAELEHRNWSKGIFSNASYKFTIPTN
ShDODAa2 LHVALGASGENSKAELVHRSWDHGTMSYASYKFT-AVS
****:**:**::****.**.*.:* :* ****** ..
N State in DODAα2
N State in DODAα1
N Alt state/state in ancestor
Convergent substitution in site
Divergent substitution in site
Unique substitution in site

Table S1: Information on primers employed in this work**.** Appropriate overhangs were added in the primers for cloning and assembling into the part plasmid entry vector pYTK001.

Name	Sequence	Gene product
yGG-LEU2 5' forward	CATAAATACCTTTCAAGC T	LEU2 integration
yGG-LEU2 5' reverse	TACAATCCTTGCCCGTGATG	LEU2 integration

Table S2: Information on Saccharomyces cerevisiae strains constructed for this work. The yeast strain yHS023 (Guerrero-Rubio et al., 2019) was employed as parental strain to integrate DODA coding sequence at the LEU2 locus. Node numbers in sequence type correspond to the labels in Figure 2a.

Strain ID	Gene ID	Sequence type	Parental strain	Marker
yHS023	Beta vulgaris CYP76AD6	Extant gene	BY4741	URA3

**Inferred ancestral samples**				
**Strain ID**	**Gene ID**	**Sequence type**	**Parental strain**	**Marker**
yPH01	amaDODAa1_a2_a4_anc_MAP	MAP, node 8	yHS023	URA3/LEU2
yPH02	amaDODAa1_a2_a4_anc_AltAll	AltAll, node 8	yHS023	URA3/LEU2
yPH03	amaDODAa1_a4_anc_MAP	MAP, node 9	yHS023	URA3/LEU2
yPH04	amaDODAa1_a4_anc_AltAll	AltAll, node 9	yHS023	URA3/LEU2
yPH05	amaDODAa4_anc_MAP	MAP, node 11	yHS023	URA3/LEU2
yPH06	amaDODAa4_anc_AltAll	AltAll, node 11	yHS023	URA3/LEU2
yPH07	amaDODAa1_anc_MAP	MAP, node 10	yHS023	URA3/LEU2
yPH09	amaDODAa1_anc_AltAll	AltAll, node 10	yHS023	URA3/LEU2
yPH10	amaDODAa2_anc_MAP	MAP, node 12	yHS023	URA3/LEU2
yPH11	amaDODAa2_anc_AltAll	AltAll, node 12	yHS023	URA3/LEU2
yPH12	acha_amaDODAa1_anc_MAP	MAP, node 3	yHS023	URA3/LEU2
yPH13	acha_amaDODAa1_anc_AltAll	AltAll, node 3	yHS023	URA3/LEU2
yPH14	coreDODAa1_grade_anc_AltAll	MAP, node 1	yHS023	URA3/LEU2
yPH15	coreDODAa1_nograde_anc_AltAll	AltAll, node 1	yHS023	URA3/LEU2
yPH16	coreDODAa1_nograde_anc_MAP	MAP, node 1	yHS023	URA3/LEU2
yPH17	cary_acha_amaDODAa1_anc_MAP	MAP, node 2	yHS023	URA3/LEU2
yPH18	cary_acha_amaDODAa1_anc_AltAll	AltAll, node 2	yHS023	URA3/LEU2
yPH19	stegnoDODAa1_anc_MAP	MAP, node 7	yHS023	URA3/LEU2
yPH20	stegnoDODAa1_anc_AltAll	AltAll, node 7	yHS023	URA3/LEU2
yPH21	macau_stegnoDODAa1_anc_MAP	MAP, node 6	yHS023	URA3/LEU2
yPH22	macau_stegnoDODAa1_anc_AltAll	AltAll, node 6	yHS023	URA3/LEU2
yAT033	GiDODA_a1_a2_limae_anc_MAP	MAP, node 4	yHS023	URA3/LEU2
yAT034	GiDODA_a1_a2_limae_anc_AltAll	AltAll, node 4	yHS023	URA3/LEU2
yAT035	GiDODA_a1_a2_anc_MAP	MAP, node 5	yHS023	URA3/LEU2
yAT036	GiDODA_a1_a2_anc_AltAll	AltAll, node 5	yHS023	URA3/LEU2
yAT037	GiDODA_a2_anc_MAP	MAP, node 17	yHS023	URA3/LEU2
yAT038	GiDODA_a2_anc_AltAll	AltAll, node 17	yHS023	URA3/LEU2
yAT039	GiDODA_a1_anc_MAP	MAP, node 13	yHS023	URA3/LEU2
yAT040	GiDODA_a1_anc_AltAll	AltAll, node 13	yHS023	URA3/LEU2
yAT041	GiDODA_a1_port_anc_MAP	MAP, node 16	yHS023	URA3/LEU2
yAT042	GiDODA_a1_port_anc_AltAll	AltAll, node 16	yHS023	URA3/LEU2
yAT043	GiDODA_a1_raph_anc_MAP	MAP, node 14	yHS023	URA3/LEU2
yAT044	GiDODA_a1_raph_anc_AltAll	AltAll, node 14	yHS023	URA3/LEU2
yAT045	GiDODA_a1_raph_no_kew_anc_MAP	MAP, node 15	yHS023	URA3/LEU2
yAT046	GiDODA_a1_raph_no_kew_anc_AltAll	AltAll, node 15	yHS023	URA3/LEU2

**Extant DODA samples**				
**Strain ID**	**Gene ID**	**Sequence type**	**Parental strain**	**Marker**
ySG063	Suaeda_maritima@62222	DODAα2	yHS023	URA3/LEU2
ySG064	Suaeda_maritima@56170	DODAα1	yHS023	URA3/LEU2
ySG065	Spinacia_oleracea@Spo27230	DODAα4	yHS023	URA3/LEU2
ySG066	Spinacia_oleracea@Spo27232	DODAα2	yHS023	URA3/LEU2
ySG067	Spinacia_oleracea@XM_021991321.1_cds_XP_021847013.1_1	DODAα1	yHS023	URA3/LEU2

ySG068	Oxybasis_rubra@6048	DODAα2	yHS023	URA3/LEU2
ySG069	Oxybasis_rubra@51962	DODAα1	yHS023	URA3/LEU2
ySG070	Extriplex_californica@28742	DODAα2	yHS023	URA3/LEU2
ySG071	Extriplex_californica@9479	DODAα1	yHS023	URA3/LEU2
ySG072	Eokochia_saxicola@53502	DODAα2	yHS023	URA3/LEU2
ySG073	Eokochia_saxicola@70762	DODAα1	yHS023	URA3/LEU2
ySG074	Froelichia_latifolia@26247	DODAα4	yHS023	URA3/LEU2
ySG075	Nitrophila_occidentalis@15180	DODAα4	yHS023	URA3/LEU2
ySG076	Chenopodium_quinoa@AUR62006948	DODAα4	yHS023	URA3/LEU2
ySG077	Amaranthus_tricolor@18737	DODAα1	yHS023	URA3/LEU2
ySG078	Amaranthus_tricolor@KP165399.1_cds_AJW81119.1_1	DODAα2	yHS023	URA3/LEU2
ySG079	Gossypianthus_lanuginosus@40105	DODAα1	yHS023	URA3/LEU2
ySG080	Gossypianthus_lanuginosus@16893	DODAα2	yHS023	URA3/LEU2
ySG081	Tidestromia_lanuginosa@4485	DODAα4	yHS023	URA3/LEU2
ySG082	Tidestromia_lanuginosa@35931	DODAα2	yHS023	URA3/LEU2
ySG083	Tidestromia_lanuginosa@35934	DODAα2	yHS023	URA3/LEU2
ySG084	Glotyphylum_uncatum@DN5796	DODAα1	yHS023	URA3/LEU2
ySG085	Glotyphylum_uncatum@DN16698	DODAα2	yHS023	URA3/LEU2
ySG086	Mirabilis_jalapa@KM502867.1_cds_AJD87536.1_1	DODAα2	yHS023	URA3/LEU2
ySG087	Mirabilis_jalapa@KM502866.1_cds_AJD87535.1_1	DODAα1	yHS023	URA3/LEU2
ySG088	Boerhavia_purpurascens@55323	DODAα2	yHS023	URA3/LEU2
ySG089	Boerhavia_purpurascens@57223	DODAα1	yHS023	URA3/LEU2
ySG090	Acleisanthes_chenopodioides@53954	DODAα2	yHS023	URA3/LEU2
ySG091	Acleisanthes_purpusiana@35413	DODAα1	yHS023	URA3/LEU2
ySG092	Acleisanthes_purpusiana@47741_ED	DODAα2	yHS023	URA3/LEU2
ySG093	Acleisanthes_purpusiana@48633	DODAα2	yHS023	URA3/LEU2
ySG094	Anisomeria_littoralis@3120	DODAα2	yHS023	URA3/LEU2
ySG095	Anisomeria_littoralis@57030	DODAα1	yHS023	URA3/LEU2
ySG096	Rivina_humilis@15447	DODAα1	yHS023	URA3/LEU2
ySG097	Rivina_humilis@420	DODAα2	yHS023	URA3/LEU2
ySG098	Montia_chamissoi@5525	DODAα1	yHS023	URA3/LEU2
ySG099	Montia_chamissoi@34252	DODAα2	yHS023	URA3/LEU2
ySG100	Talinum_paniculatum@39504	DODAα1	yHS023	URA3/LEU2
ySG101	Talinum_paniculatum@6206	DODAα2	yHS023	URA3/LEU2
ySG102	Tacinga_lilae@22876	DODAα1	yHS023	URA3/LEU2
ySG103	Tacinga_lilae@80758	DODAα2	yHS023	URA3/LEU2
ySG104	Pterocactus_tuberosus@67194	DODAα1	yHS023	URA3/LEU2
ySG105	Pterocactus_tuberosus@73720	DODAα2	yHS023	URA3/LEU2
ySG106	Echinopsis_aurea@71662	DODAα2	yHS023	URA3/LEU2
ySG107	Echinopsis_aurea@86484	DODAα1	yHS023	URA3/LEU2
ySG108	Talinopsis_frutescens@1849	DODAα1	yHS023	URA3/LEU2
ySG109	Talinopsis_frutescens@35327	DODAα2	yHS023	URA3/LEU2
yRG009	Beta vulgaris DODAα1	DODAα1	yHS023	URA3/LEU2
yRG010	Beta vulgaris DODAα2	DODAα2	yHS023	URA3/LEU2
yRG006	Beta vulgaris DODAα4	DODAα4	yHS023	URA3/LEU2
yAT011	Mesembryanthemum crystallinum DODAα1	DODAα1	yHS023	URA3/LEU2
yRG008	Mesembryanthemum crystallinum DODAα2	DODAα2	yHS023	URA3/LEU2
yAT001	Carnegiea gigantea DODAα1	DODAα1	yHS023	URA3/LEU2

yAT002	Carnegiea gigantea DODAα2	DODAα2	yHS023	URA3/LEU2
